# Developing and testing an environmental economics approach to the valuation and application of urban health externalities

**DOI:** 10.3389/fpubh.2023.1070200

**Published:** 2023-02-17

**Authors:** Eleanor Eaton, Alistair Hunt, Daniel Black

**Affiliations:** ^1^Department of Economics, University of Bath, Bath, United Kingdom; ^2^Population Health Sciences, Bristol Medical School, University of Bristol, Bristol, United Kingdom; ^3^Daniel Black + Associates | db+a, Bristol, United Kingdom

**Keywords:** health, environmental economics, decision-making, urban development, planning

## Abstract

**Background:**

Poor quality urban environments have substantial impacts on public and planetary health. These costs to society are not readily quantifiable and remain largely external to mainstream measures of progress. Methods for accounting for these externalities exist, but their effective application is in development. Yet there is an increasing urgency and demand given the profound threats to quality of life both now and in the future.

**Methods:**

We combine data from a series of systematic reviews of the quantitative evidence linking characteristics of the urban environment with health consequences and the economic valuation of these health impacts from a societal perspective within a spreadsheet-based tool. The tool–named HAUS–allows the user to estimate the health impacts of changes in urban environments. The economic valuation of these impacts in turn facilitates the use of such data in broader economic appraisal of urban development projects and policies.

**Findings:**

Using the Impact-Pathway approach, observations of a variety of health impacts associated with 28 characteristics of the urban environment are applied to forecast changes in cases of specific health impacts that result from changes in urban contexts. Unit values for the societal cost of 78 health outcomes are estimated and incorporated in the HAUS model in order to allow the quantification of the potential effect size of a given change in the urban environment. Headline results are presented for a real-world application in which urban development scenarios that have varying quantities of green space are evaluated. The potential uses of the tool are validated *via* formal semi-structured interviews with 15 senior decision-makers from the public and private sectors.

**Interpretation:**

Responses suggest that there is significant demand for this kind of evidence, that it is valued despite the inherent uncertainties, and has a very wide range of potential applications. Analysis of the results suggest expert interpretation and contextual understanding is critical for the value of evidence to be realized. More development and testing is needed to understand how and where it may be possible to apply effectively in real world practice.

## 1. Introduction

The quality of our urban environments impacts substantially on human and planetary health. Air pollution, lack of access to nature, low availability of healthy food and drink, and inactive lifestyles all contribute to non-communicable diseases (NCD) such as obesity, diabetes, respiratory illness, anxiety and depression. Together these NCDs make up 89% of deaths in the UK, most of which are seen as preventable (1, 2). Socio-economic pressures compound these impacts significantly (3). Climate change may also act as a stress multiplier in urban centers, exacerbating existing problems such as overheating and flooding (4).

Some estimates of economic costs have been made that attribute costs to these risk factors in the urban environment. Though disconnected and overlapping, they do give a sense of the scale of the challenges. For example, income inequality, which is strongly linked to poor quality urban environments through quality of housing and accessibility of green infrastructure, has been estimated to result in productivity losses of £31–33 billion per year (3). A separate estimate suggested low quality property and neighborhoods in England cost the UK National Health Service £1.4 billion annually in treatment provision (5). Obesity, which has multiple risk factors, including obesogenic environments and “food deserts” (i.e., lack of healthy food in a local area), costs an estimated £27bn per year due to its negative effects on productivity, earnings, and welfare payments (2). Costs from climate change are also likely to be very substantial–for example, one estimate suggests it will add £120bn to property insurance costs by 2040 and have adverse impacts on human health through overheating in buildings, storms and flooding (6).

Alongside these estimates of financial costs associated with the urban environment there is a small literature that recognizes the non-market dimension to welfare loss attributable to components of this environment. The use of economic valuation approaches in measuring, and accounting for, non-market environmental and social “goods and services,” including human health outcomes, has a substantial history (7). However, its integration in to mainstream decision-making has been slow for a number of reasons, not least the considerable challenge of quantifying intangible aspects of health in welfare terms (8, 9). The exception to this is in the air pollution context–an environmental hazard that is most severe in urban areas where population density is highest. For example, the UK Government estimates average damage costs–including both market- and non-market health costs of air pollution associated with particulate matter, nitrogen dioxide, ammonia, volatile organic compounds and sulfur dioxide (10). These damage costs are disaggregated by rural and urban location, the urban locations being further disaggregated by size of conurbation (11).

This lack of uptake does not appear to imply a lack of appetite for non-market valuation. For example, a series of 30 interviews with senior decision-makers from public and private sectors suggest that there is a strong desire for more comprehensive, approaches to valuation of health in urban areas (10). These interviews highlight a range of potential areas of application, including: government investment programs, land valuation, private sector investment and planning decisions (12, 13).

There currently exist a number of tools that generate quantitative estimates of health impacts that may be expected to result from a local policy or intervention within the urban context. WHO Europe has developed the Health Economic Assessment Tool (HEAT) for assessing changes in cycling and walking provision and patterns, using estimates of reductions in mortality as a benefit of increased active travel (14). The tool uses a Value of a Statistical Life (VSL) to estimate the value of changes to mortality; morbidity is excluded. The ITHIM model, developed in the UK and applied there and in the US, has also been used to estimate the health impacts of transport interventions, using productivity losses and treatment costs of illness to estimate the value of attributable changes to mortality and morbidity (15).

Additional social valuation tools methods that incorporate health impacts have emerged in the UK since the United Kingdom 2012 Public Services (Social Value) Act (16), that has as a legal requirement consideration of wider social, economic and environmental benefits additional to financial profit. These include the UK Social Value Bank (17), the National TOMs framework (18) and the Manchester Cost Benefit Analysis tool (19). Health is typically just one of many outcomes included in these models, such as employment, volunteering, crime and perceptions of local environment. These models do not offer a method for estimating potential changes to health, but rather offer a database of unit values to help policy makers estimate the social value of public sector investment such as neighborhood improvements which may impact on health. Health in these models is defined in terms of self-rated life satisfaction rather than by individual morbidity end-points. For example, unit values are given in terms of episodes of hospital attendance rather than cases of asthma. Mortality is not normally included.

We have created a tool for urban planners which allows the user to consider all determinants of health which relate to new urban housing developments. In doing so, we address gaps identified above in existing tools by estimating and valuing changes to health risk both in terms of morbidity and mortality and address a wide range of environmental determinants of health which have been linked with urban development. We provide a resource of unit costs for 76 health outcomes, disaggregated so that they can be attributed across multiple agencies from a societal perspective.

This study adopts an approach to quantification based on the Impact-Pathway method which uses known pairings in the published literature between individual characteristics of the environment, such as PM_2.5_ air pollution, and specific observed health outcomes to forecast changes in cases of morbidity and mortality resulting from a change in the environment. These health cases can then be monetized and aggregated to estimate the social value of an intervention (20, 21). We extend this approach to a wider range of environmental determinants of health than has been attempted previously. In doing so we utilize the findings of a series of systematic reviews on the quantitative relationships between characteristics of the urban environments and health outcomes, and evidence on the economic welfare valuation of the identified health outcomes (22, 23). The innovation is not in the modeling *per se*, but in the integration of multiple approaches, including: the systematic review of urban-health evidence, an environmental economics approach to valuation of urban health externalities, and the validation of our approach with potential end users.

This paper first outlines the approach taken to express quantitative health impacts of the urban environment in economic terms. We then present indicative findings from an application of the model in the context of an urban regeneration plan in Bristol, UK. We review these findings, reflecting critically on the current limitations to this modeling as well as its possibilities.

## 2. Methods and materials

### 2.1. Definition of the urban form elements

In principle, our model is intended to encompass as comprehensive a range of characteristics of the urban environment as possible, thereby ensuring that consideration of any associated impacts on health in decisions relating to urban development are as complete as possible. In order to achieve this the extent was scoped in the first instance by adopting the categories defined by the Health Map, (24), a classification of the health determinants associated with the planning of human settlements published by the Royal Society for the Promotion of Public Health, and offering a comprehensive coverage of socio-environmental issues relating to urban planning and design. This classification was validated against similar classifications assembled in five other checklists including: Public Health England's Topics (25), Vancouver Healthy Toolkit (26), BREEAM Communities (27), HUDU Rapid HIA (28) and Egan Review (29) (see Supplementary material).

**Figure 1:**
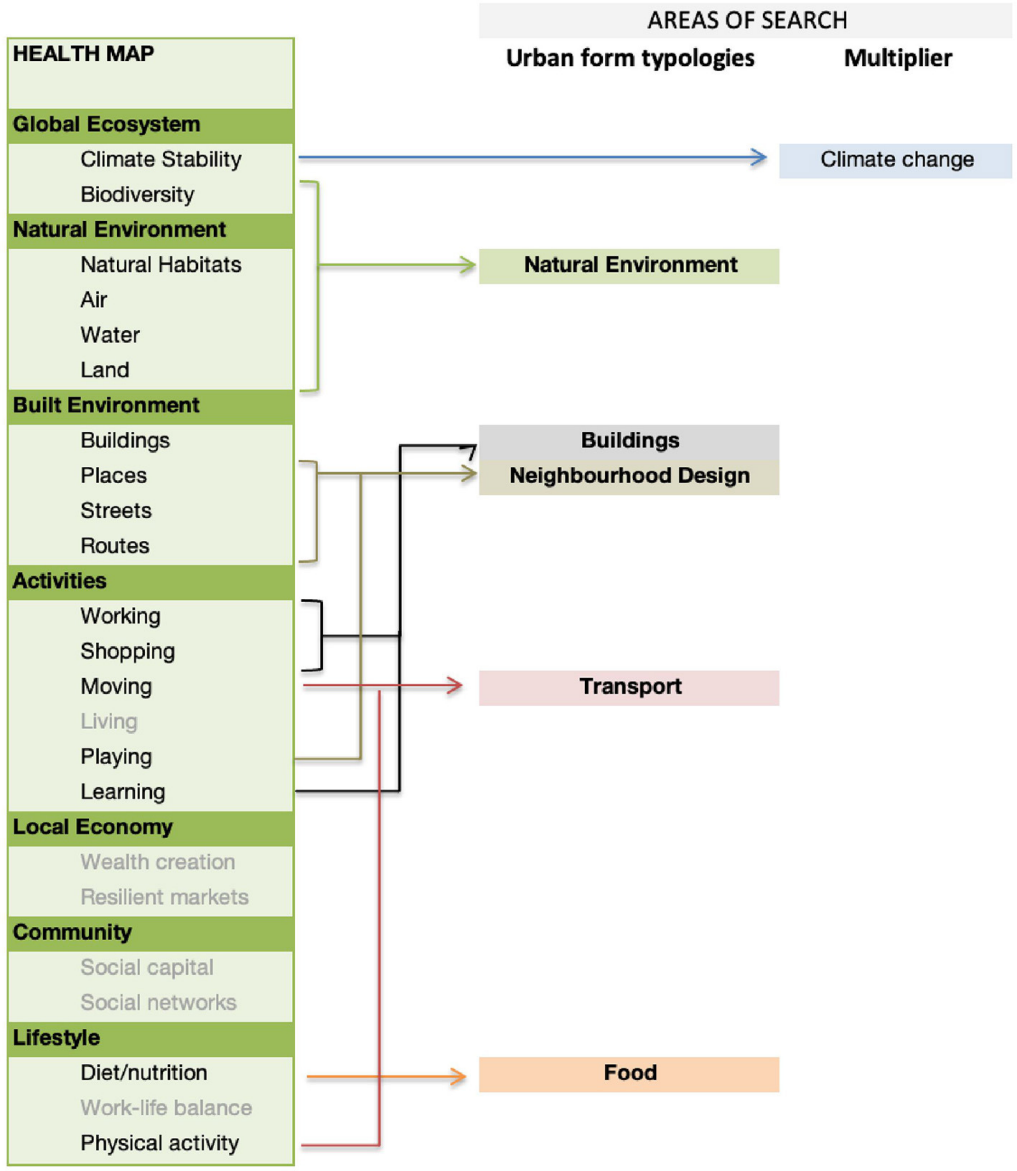
Identification of five main search areas (of urban form typologies), derived from the Health Map.

We then grouped the 23 aspects of the urban environment in the Health Map into five main “typologies” of urban form (or areas of search): natural environment, buildings, neighborhood design, transport and food; climate change was categorized as a “multiplier” of each element of urban form (Figure 1). Six areas from the Health Map were excluded as they are not explicitly related to elements of the urban form: living, wealth creation, resilient markets, social capital, social networks, work-life balance (as shown in gray in Figure 1).

### 2.2. Identification of health impacts

We identify the individual health impacts associated with the five urban form typologies on the basis of the systematic reviews previously undertaken that use these classifications (22, 23). From the initial five search areas (Figure 1), the evidence derived through the systematic reviews resulted in a slightly changed list of urban form typologies. Buildings, Natural Environment, Climate Change and Transport remain the same, but Neighborhood Design and Food are combined into Community Infrastructure, and we use an extra category of Socio-economics to include elements such as affordability, living in areas of high poverty and renting vs. home ownership.

We understand health impacts to include mortality and non-communicable disease, including physical and mental illness, congenital deformities, injuries from road traffic and domestic accidents, loss of physical functioning and limits to daily activities, symptoms of illness such as wheezing, behaviors such as activity or diet, mental illness, obesity, and measures of wellbeing such as life satisfaction scores. We also include upper and lower respiratory tract infections, including colds and flu. We do not include dental problems, sexually transmitted disease, memory problems, educational attainment, or injuries from assault, all of which are less directly associated with the elements of the urban form that we have identified.

**Figure 2:**
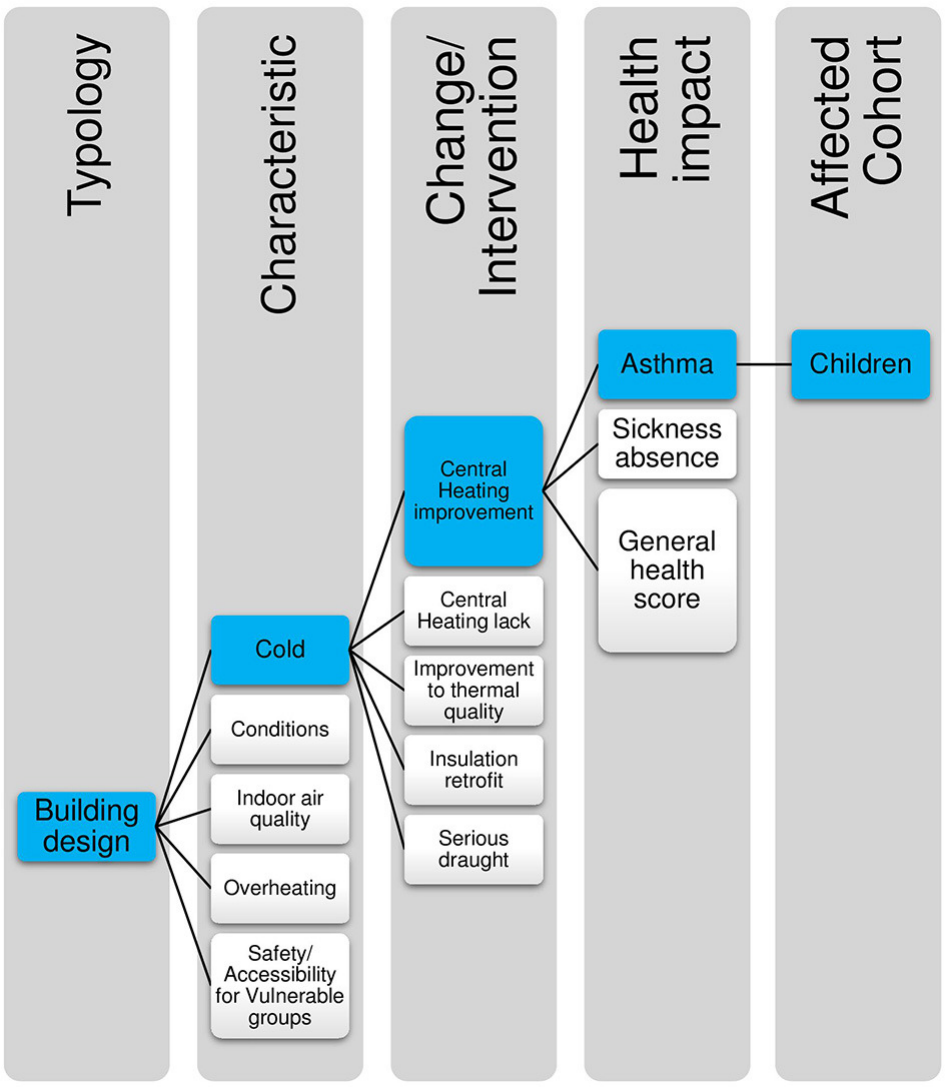
Illustration of how impact pathways are defined: highlighted in blue is the specific pathway for central heating improvements and asthma in children.

The epidemiological literature reported in the systematic reviews (22, 23) allow us to identify 170 urban environment characteristic-health impact pathways that observe a causal path from a specific environmental change (such as air pollution or increased green space) to a health outcome (such as increased risk of asthma or diabetes). These are listed in the Supplementary material. An example of one such pathway is presented in Figure 2.

Impact pathways are defined here as estimates of the magnitude of effect that a specific change within a single characteristic of the urban environment may have on a specific health outcome. These impact pathways are defined by the specific parameters from the original source study or studies, and include detail of the specific environmental change, the size and scale of the effect, the population demographics of the original study, and the individual health outcome. Where multiple pieces of evidence exist relating to the same environmental feature and the same health outcome, for example in levels of PM_2.5_ in air and asthma in children, data was selected on the basis of strength of evidence, robustness, and applicability to the specific UK housing context.

Evidence for change defined in the impact pathway is expressed in the form of dose-response; i.e., for a specific change in environmental characteristic, a quantitative measurable change in health status is observed as a change in the risk (known as “odds”) of illness. The epidemiological evidence is primarily described in terms of linear relationships. We judge that, given the wide range of real-world contexts, this is unlikely always to be the case. Consequently, our model outputs should be regarded as approximations of the size of health changes associated with changes in the urban environment.

In the HAUS model the aim is to identify environmental changes at an intervention specific level, so that it is possible to compare the efficacy of alternative interventions. Impact pathways are highly specific, replicating the individual parameters of the original study. For example, Figure 2 indicates how the impact pathway of Central heating improvements > asthma in children sits within the typology of Building Design and the Characteristic of Cold.

### 2.3. Specification of the model

In this paper we develop an economic tool that enables stakeholders to quantify the impact on population health of a specific intervention or policy relating to the environment in urban environments.

The tool is known as the Health Appraisal for Urban Systems tool (or HAUS for short). HAUS has three key features:

- It synthesizes the available evidence to allow policy makers to access data on measurable, quantifiable changes to health which have been associated with the urban environment;
- It enables users to estimate the potential magnitude of impact which a specific intervention may have on the health of residents;
- It offers a method for valuing these health impacts, for use in cost-benefit, or cost-effectiveness analysis of alternative projects, policies or other intervention forms.

It is therefore intended for use as a tool to support scenario appraisal and to inform broader conversations around prioritization in health in urban development. The HAUS tool covers non-communicable disease in all populations in the UK, including older adults and children–categories not disaggregated within existing tools.

It is capable of estimating effects at the neighborhood scale, and can be extended to take into account different population sizes impacted but is not designed to be used to estimate effects on an individual or a single family group.

### 2.4. Structure of the health appraisal for urban systems tool

**Figure 3:**
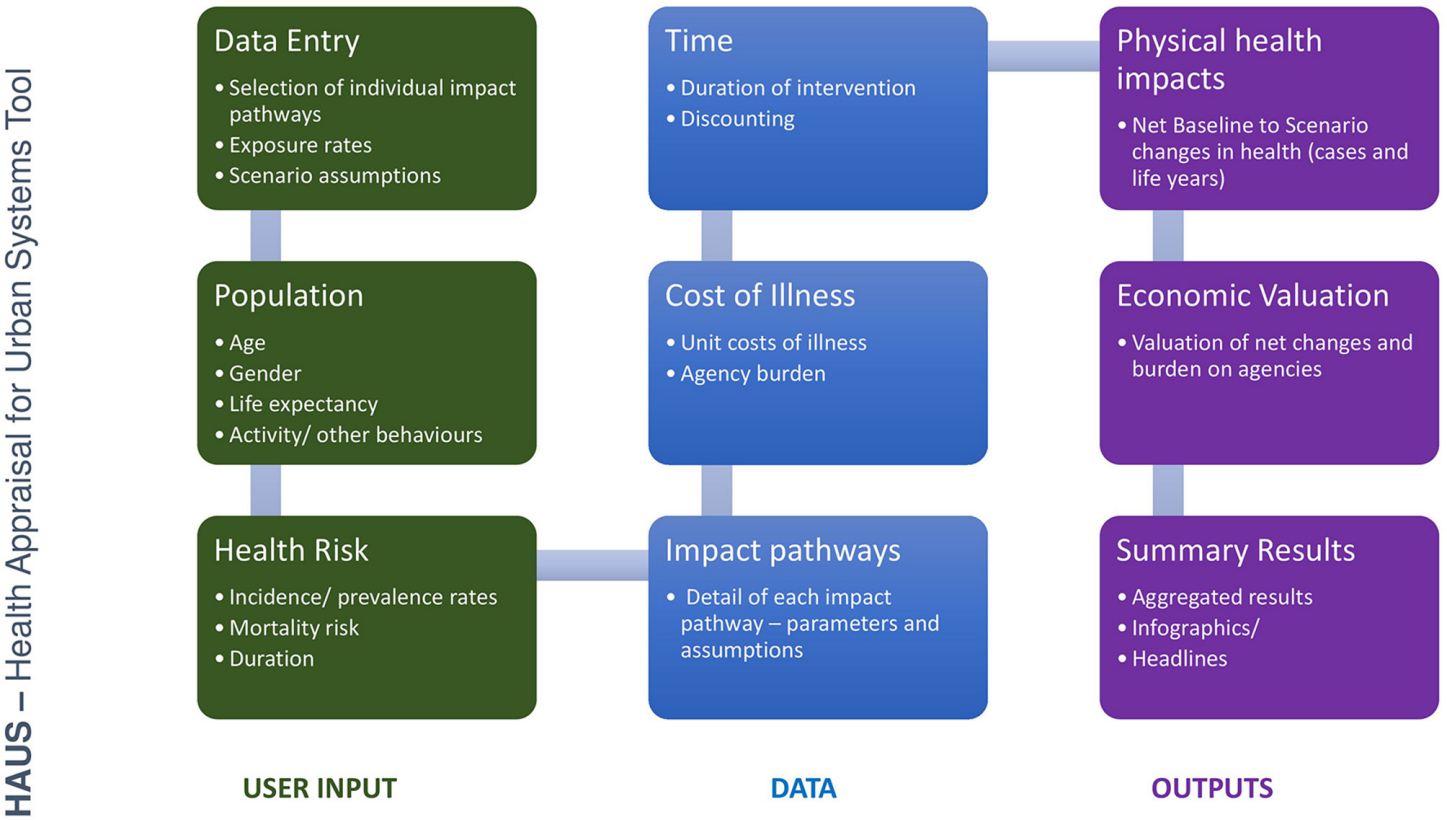
Illustration of the structure of the Health Appraisal for Urban Systems (HAUS) tool.

The HAUS tool is initially a spreadsheet-based system, for ease of use and software availability, created in Excel software (30). The structure of the tool is set out in Figure 3.

The tool includes the following:

- User input sheets: allowing the user to identify key assumptions around each scenario for assessment, such as population size and level of exposure to each specific environmental feature. In the absence of user-defined data, the tool specifies default settings for a given population.
- Data sheets: Data on demographic profile of the affected population, individual health end-point risk, and unit costs of illness.
- Outputs: Detailed comparisons of results and valuations of health impacts, and a dashboard which allows the user to quickly identify headline information.

**Figure 4:**
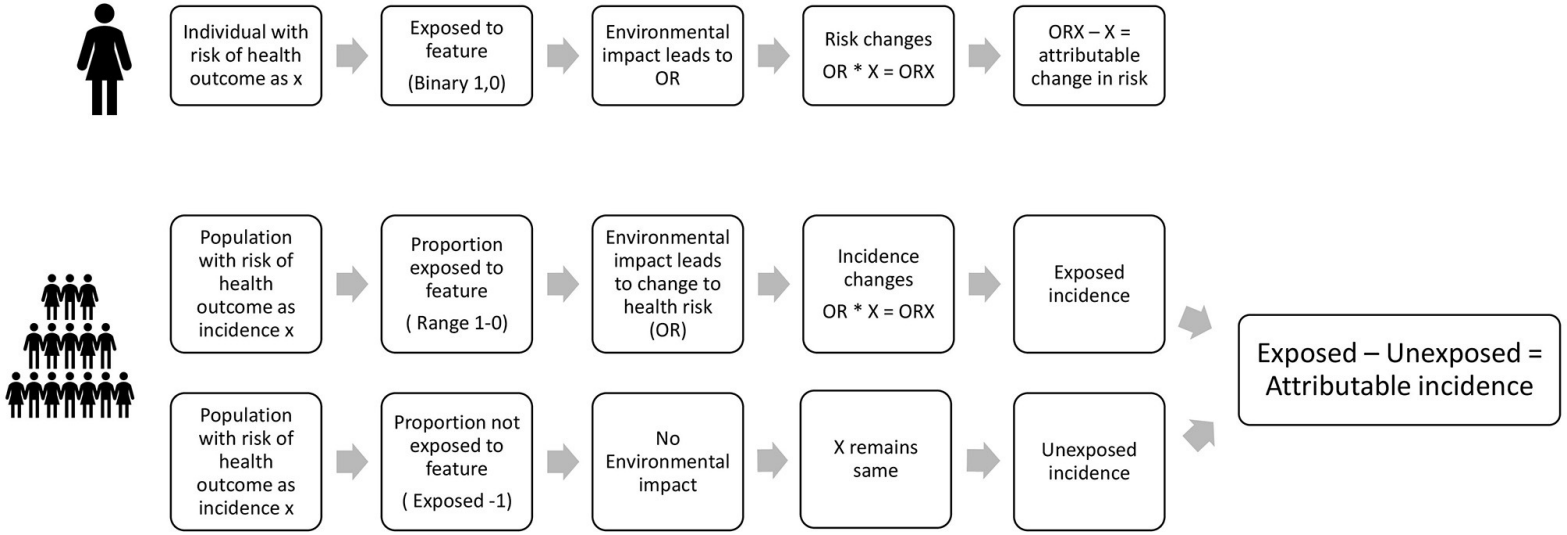
Estimation of attributable changes in health in HAUS for an individual and for a population.

The estimation process undertaken within the HAUS tool is illustrated in Figure 4. We present health impacts in terms of estimated attributable changes to cases of illness, deaths, years of illness and years of premature life lost.

These are derived in the following way:

Each individual has an existing risk of contracting a particular illness, for example, asthma, or diabetes. We assume that if they are exposed to a change in environmental conditions, this may alter their odds, or risk, of contracting that disease. The difference between their original risk of disease, and the new odds of the disease is the amount of risk which can be attributed to the environmental change. This principle is applied in the same way to a whole population likely to be impacted by the change–the population being defined in the impact-pathway specification. Estimation of the risk of being affected by the illness in question (e.g., asthma, diabetes, etc.) is therefore made in relation to the baseline, i.e., the existing incidence rate of the illness in the population. When part of the population is exposed to a change in environment, we measure the attributable change in incidence by comparing the incidence in the exposed population to the incidence in the unexposed population.

In the HAUS tool, we therefore apply changes in odds or risk of disease which have been observed in the epidemiological literature as being significantly associated with a change in a specific environmental characteristic, or feature, to the exposed population. This method can be delineated more precisely using a notational system, described here for mortality and morbidity respectively.

### 2.5. Estimation of mortality effect

We calculate mortality in terms of two metrics: numbers of attributable deaths (*Dattributed*) and attributable premature years of life lost (*YLLattributed*). Deaths and premature life years lost here are defined as statistical lives and statistical life years lost–representing the sum of many small numbers of risks of life lost, rather than individual people.

#### 2.5.1. Estimation of attributable deaths

We assume that the annual expected number of deaths in a given population (*De*) can be calculated by multiplying the number of people in the intervention area *(n)* by an average annual mortality rate (*MRlit*) in the baseline literature, e.g., national demographic statistics. We then estimate the proportion of the number of people exposed to a hazard as *Pe* and the number of unexposed people as *Pu*, i.e., *(1-Pe)*.

We assume that the mortality rate for the exposed populations is affected by the odds ratio, *OR*, associated with the hazard so that *MRe* = (MRlit^*^OR). The OR is derived from the specific impact pathway. The following conditions therefore hold:


$$\begin{array}{l} \;MRu=MRlit\\ \;MRe=MRlit^{*}OR\\ \;Pe=ne/n\\ \;Pu=1-Pe\\ \;De=MRe^{*}(n^{*}Pe)\\ \;Du=MRu^{*}(n^{*}Pu)\\ Dattributed=(De+Du)-Du \end{array}$$

where:
*MRlit* = *mortality rate in the assessed population derived from literature (for a specified age range)*
*MRe* = *mortality rate in the exposed population*
*MRu* = *mortality rate in the unexposed population*
*OR* = *Odds Ratio for change in mortality derived from Impact-Pathway*
*n* = *the population assessed in HAUS*
*ne* = *the number of exposed people in the population*
*nu* = *the number of unexposed people in the population*
*Pe* = *Proportion of the assessed population that is exposed*
*Pu* = *proportion of the assessed population that is not exposed*
*De* = *deaths in the assessed population with exposure*
*Du* = *deaths in the assessed population without exposure*
*Dexpected* = *expected deaths in the population*
*Dattributed* = *deaths attributed to the exposure assessed in HAUS*.

#### 2.5.2. Estimation of attributable life years lost

We calculate the number of preventable life years lost (*YLL*) as following:

Life years (*LY*) are the sum of the expected years of life in the sample population *n*.

(This is calculated on the basis of: *n* in each age year ^*^ life expectancy for each age year).

Premature life years lost attributed to the exposure (*YLLattributed*) are calculated on the basis of average Life Years ($\bar{LY}$) multiplied by the number of deaths estimated in the exposed and unexposed populations.

Attributable premature years of life lost: *YLLattributed* = *YLLe–YLLu*


$$\begin{array}{l} \;LY={\sum^{\text{​}}}_{i=1}^{n}LE\\ \;\bar{LY}=\frac{LY}{n}\\ \;YLLe={\bar{LY}}^{*}De\\ \;YLLu={\bar{LY}}^{*}Du\\ YLLattributed=YLLe-YLLu \end{array}$$

where:
*LE* = *life expectancy in years for each age group in the population*
*LY* = *Sum of life expectancy in years*

$\bar{LY}=\;$ *Average life expectancy in years*
*De* = *deaths in the assessed population with exposure*
*Du* = *deaths in the assessed population without exposure*
*YLLe* = *years of life lost in the assessed population with exposure*
*YLLu* = *years of life lost in the assessed population without exposure*
*YLLattributed* = *years of life lost attributed to the exposure assessed in HAUS*.

#### 2.5.3. Estimation of morbidity effect

We estimate morbidity effects in terms of two metrics: attributable cases of illness (*Cattributed)* and years with illness (*YLDattributed*).

##### 2.5.3.1. Estimation of attributable cases of illness

We assume that the expected number of cases of illness in the population (*Cexpected*) can be calculated by multiplying the number of people in the intervention area (*n*) by an incidence rate (*IRlit*). We again forecast the proportion of the number of people exposed to a hazard as *Pe* and the number of unexposed people as *Pu (1-Pe)*. We assume that the incidence rate for exposed populations is determined by the odds ratio associated with the hazard *OR* so that *IRe* = (IR^*^OR).

Cases Exposed *Ce* = (n^*^Pe)^*^(IRe)
Cases Unexposed *Cu* = (n^*^(1–Pe))^*^IRu)

Attributable cases of illness: *Cattributed* = *(Cu*+*Ce)-Cu*

*IRu* = *IRlit*
*IRe* = IRlit^*^OR
*Pe* = *n*e/*n*
*Pu* = *1 – Pe*
*Ce* = *IRe* ^*^ (n^*^Pe)
*Cu* = *IRu* ^*^ (n^*^Pu)

where:
*IRlit* = *Incidence rate in the assessed population derived from literature (for a specified age range)*
*IRe* = *Incidence rate in the exposed population*
*IRu* = *Incidence rate in the unexposed population*
*OR* = *Odds Ratio for change in risk of illness derived from Impact-Pathway*
*n* = *the population assessed in HAUS*
*ne* = *the number of exposed people in the population*
*nu* = *the number of unexposed people in the population*
*Pe* = *Proportion of the assessed population that is exposed*
*Pu* = *proportion of the assessed population that is not exposed*
*Ce* = *Cases of illness in the assessed population with exposure*
*Cu* = *Cases of illness in the assessed population without exposure*
*Cexpected* = *Cases of illness expected in the population*
*Cattributed* = *Cases of illness attributed to the exposure assessed in HAUS*.

##### 2.5.3.2. Estimation of attributable years spent with illness

We calculate the number of years with illness or disability (*YLD*) based on the sum of years of life expectancy (*LE*) in the sample, and the expected duration of the illness (*Tsick*) capped with Life Expectancy (*LE*) so that *Tsick* cannot exceed *LE* for any individual:

Attributable years lived with illness *YLDattributed* = Cattributed^*^YLD


$$YLD=\sum_{i=1}^{n}\{\begin{array}{c} Tsick\;if\;Tsick>LE\\ LE\;if\;Tsick<LE \end{array}$$

where:
*YLD* = *Sum of years of life spent with illness or disability (or remaining life expectancy) in population affected by the illness*
*LE* = *Life expectancy in years for each age group in the population*
*Tsick* = *Average duration of a case of illness, derived from the literature*
*YLDattributed* = *years of illness attributed to the exposure assessed in HAUS*.

#### 2.5.4. Estimation of time effects

We calculate the sum of attributable cases or deaths over time applying the number of years of the project as a simple linear multiplier, assuming that mortality rates, morbidity incidence rates and risk ratios are linear and do not change over time.

The total effect of an intervention (*Total effect)* over the duration, *Tintervention*, is therefore as follows:


$$Total\;Effect=Tintervention*\{\begin{array}{c} Dattributed\\ Cattributed \end{array}$$

We assume that there is a lag between a change in environment and full health effect of 5 years, so that in the first year only a 20% of the full effect is estimated, 40% in the second year, and so on, increasing by 20% each time. The total effects are also capped, so that we only include health effects expected within the lifetime of the project, set at 25 years.

Life expectancy data and population demographics are derived from Office of National Statistics statistical datasets for the reference year 2019 (31).

Information on disease incidence rates and mortality rates were derived from a number of sources, including mortality data from the Office for National Statistics (32), Hospital Episode statistics from NHS Digital (33), and specific disease incidence from the Global Burden of Disease Study (34). Information on wellbeing and mental health, activity levels and other behaviors are derived from the Health Survey for England (35).

Wherever possible, incidence rates are identified as relating to the UK population for 2019, but where UK data has not been available we have referred to data for England.

### 2.6. Estimation of value of health impacts

Economic appraisal is an integral part of decision making when policy makers seek to find the most efficient use of resources. Rooted in welfare economics, economic appraisal attempts to define whether a project makes a net contribution to social welfare. At its heart are methods for quantifying and valuing changes to individuals' utility as a result of a change in health, so that these values can be used in cost-benefit analyses, for example. In this paper we value health impacts from the societal perspective, taking into account the impact of health on the individual, their family, employers, healthcare providers and the state. This approach incorporates different components of the welfare costs of illness, including direct medical and paid care expenses, indirect lost opportunity costs such as productivity and the value of informal care time, as well as a value which monetises the disutility or pain and suffering associated with disease.

The HAUS model estimates the monetary equivalent of the disutility relating to a loss of welfare associated with risks of premature death and illness. Disutility is expressed as an individual's Willingness to Pay (WTP) to avoid illness or for improvement in health and is assumed to be the sum of the observable cost of illness (lost wages and mitigation costs) and the monetary equivalent of the non-observable cost of lost utility (mortality, pain and suffering). These non-observable costs are estimated using non-market valuation methods.

Society has mechanisms for shifting many costs of illness away from the individual–i.e., *via* medical insurance and sick leave policies (36). This is particularly relevant for the UK, where most healthcare is free at the point of use. We attempt to define the societal impact of changes to health status across a population and identify where the burden of costs of illness falls. In the process of doing so, we utilize data from a range of sources including the published literature on non-market values. In this instance value transfer methods are adopted to ensure that value estimates derived in the context of previous studies are adjusted to reflect their transfer to a different context.

In order to estimate the value of identified changes in health in each impact pathway we multiply the unit values calculated for each specific health impact by the attributable health impact.

Unit values for morbidity impacts are estimated per year of ill health and per case of illness whilst unit values for mortality are estimated as the Value of a Statistical Life (VSL) and the Value of a Statistical Life Year (VSLY).

A library of reference values relating to direct and indirect costs and disutility derived from primary studies was estimated using a systematic review approach, using meta-analysis, benefits transfer techniques and quality assessment to derive reference values and ranges from the primary and secondary evidence base.

A systematic review of published literature was carried out, with additional modeling to estimate unit values for the range of health impacts included in our HAUS model. Electronic sources for peer-reviewed literature were searched, followed by reference searching. Studies were included that had clearly stated methodologies, were written in English, and which could be utilized in a UK context. The search prioritized studies from 2016 to 2020, which estimated values at an individual, per annum or per case level. Reference unit values are estimated for 76 individual health outcomes, including physical and mental illness, mortality, and health related behaviors, such as activity, obesity, and alcohol misuse.

Unit values are estimated for each health outcome in GBP £2019 (Supplementary Table 2). 2019 has been chosen as the reference year for health impacts because of the significant changes to the experience and recording of health since the COVID-19 pandemic began in the UK in March 2020. For example; we know that during this period unusual patterns occurred in expected mortality and hospital admissions, and lockdown restrictions were put in place which affected normal active behaviors (31). This may mean that data for 2020 and 2021 are atypical for use in forecasting future trends of health.

## 3. Results—Development and testing of the HAUS model

The methodological approach outlined above and informing the HAUS model has so far been tested with external practitioners in two ways: (i) by presenting illustrative findings as part of a number of interviews with public and private sector decision-makers, and (ii) *via* use of the model with case study partners, focusing specifically in the first instance on green infrastructure (this second part forms part of a wider exercise developing valuations across the full range of typologies above).

### 3.1. Interviews overview

Two rounds of semi-structured interviews were undertaken with 15 senior decision-makers from a purposive sample of the UK's main urban development delivery agencies, both public and private. Methods and findings from the interviews are to be found in separate papers (12, 13). Engagement at senior level with those who have control over key aspects of planning and development implementation—such as land disposal, investment, development delivery and planning permission—was central to the approach. Field notes of the interviewee responses to four questions on the economic valuation are included in the Supplementary material, and summary reflections provided below.

### 3.2. Case study: Frome Gateway regeneration site, Bristol, UK

#### 3.2.1. Background

Frome Gateway is a 14.7 hectare site in the center of Bristol. The site has been designated a strategically important site in need of major regeneration by Bristol City Council (37). A map of the site can be seen on the Bristol City Council website for Frome Gateway (38).The draft Local Plan set out the ambition for the site to be developed as a new mixed-use neighborhood, including around 1,000 new homes, improved access to the River Frome and existing green spaces, improved connectivity to the site generally, and improved opportunities for work and leisure (37).

HAUS was used to provide detailed information on expected health outcomes related to the scenarios under development and so to increase knowledge about the potential for environmental impacts on health. The specific objectives for evaluating the impact of changes to parks and green spaces includes the following:

1. To identify the health benefits of the parks and green spaces in Frome Gateway in a future scenario, compared with present day and alternative scenarios.
2. To identify the health benefits of providing a specific amount of additional green space in a single large unit, such as a new park.
3. To ascertain whether the same benefits as in (2) can be realized by dispersal of additional green space across the site, such as a series of small pocket parks.

#### 3.2.2. Parameters and scenario building

In order to provide comparative information, four scenarios were developed presenting alternative patterns of development for the site:

##### 3.2.2.1. Baseline scenario: Present day conditions

We assume that the existing quality, condition and area of green space, including the parks and river areas, are as the present day. The site has 2.37Ha of green space mostly contained within two parks: Riverside Park and Peel Street Open Space (39).

##### 3.2.2.2. Definition of future scenarios

1. Scenario 1: (Policy Compliant) improvements in quality of green spaces and 0.31 Ha additional green space consistent with minimum policy ambitions (37).
2. Scenario 2: Future Scenario (single open space): one additional large green space of around 1 Ha dimension, in addition to the provision of green space in Scenario 1.
3. Scenario 3: Future Scenario (dispersed open space): As Scenario 2, but green space provided by a series of small pocket parks (defined as spaces under 0.5Ha in dimension).

A project lifetime of 25 years is assumed. Effects are estimated for an area including a buffer of 300 m around the perimeter of the site, which is used to take into account effects on local communities.

#### 3.2.3. Data

Information on Green Infrastructure was derived using Natural England's Green Infrastructure Framework Map tool (40). The Normalized Difference Vegetation Index (NDVI) score for the site is assumed to be 0.15, with the NDVI for Riverside Park estimated at 0.29. NDVI is used as a measure of exposure to greenness in several of the health studies used in HAUS. NDVI uses satellite imagery to estimate the greenness of an area, with higher scores on a range of −1 to 1 indicating higher levels of greenness. NDVI can be useful as a way of determining the proximity of different types of vegetation, such as grass and trees (41).

Assumptions around environmental conditions are derived from the Development Assumptions Report, technical reports and local site visits (39, 42). Local residents' perceptions of the area, activity levels and usage of parks/open spaces were not known, so a survey of 108 residents living near to the site was carried out in 2022. The survey results provided input to the HAUS model and further contextual information for the regeneration team.

#### 3.2.4. Population

The total affected population, including those within 300 m of the site, is estimated at 9,241. We assume 3,000 residents live within the site boundary in all scenarios. This is based on the provision of around 1,000 new homes with an average occupancy of 2.5 per household, plus an additional 500 student residences.

At present only a small number of homes are present within the site boundary. For easier comparison, the baseline scenario adopts a hypothetical 3,000 residents, reflecting the projected population size in the future scenarios.

#### 3.2.5. Results

##### 3.2.5.1. Health benefits of green space at Frome Gateway: Baseline

The results for the baseline indicate that the existing green space are likely to provide a range of health benefits, especially for adults using the parks who are found to experience reductions in diabetes and reduced risk of weight gain. These are shown in Tables 1, 2. There may, however, be a negative effect from green space on risk of asthma in children, deriving e.g., from pollen: from 8 expected cases in this age group, we estimate a potential increase of 5 attributable cases per year.

**Table 1:** Attributable morbidity affected by green space–baseline.

**Characteristic**	**Environment change^a^**	**Health outcome**	**Population**	***N***	**Cases expected^b^**	**Baseline cases^c^**	**% change**
Green space	NDVI increase	Cancer (mouth and throat)	Adults > 18	6,927	2	0	0%
Green space	NDVI increase	Respiratory (asthma)	Children < 7	1,223	18	0	0%
Green space	NDVI increase	Weight gain	Children 9–12	583	216	0	0%
Green space	Proximity to green space	Respiratory (asthma)	Children 9–12	583	8	5	60%
Green space	Size of public open spaces	Diabetes	Adults >18	6,927	41	0	0%
Places to play	Park use	Diabetes	Adults >35	3,262	20	−3	−15%
Places to play	Park use	Weight gain	Adults >35	3,262	979	−127	−13%
Total				9,241			

a Specific environment related intervention, asset or hazard).

b Cases expected in same population per annum without any environmental pathways applied.

c Baseline refers to net cases in present day scenario (Attributable cases of morbidity per annum minus expected cases).

Red highlighted text denotes deterioration in health. Green text indicates improvement in health.

**Table 2:** Attributable activity, general health and wellbeing affected by green space–baseline.

**Characteristic**	**Environment change^a^**	**Health outcome**	**Population**	***N***	**Cases expected^b^**	**Baseline cases^c^**	**% change**
Green space	Proximity to green space	Activity	Adults >18	6,927	4,295	2,632	61%
Green space	Proximity to green space	Mental health	Adults >65	588	463	125	27%
Green space	Proximity to large, attractive, open space	Activity	Adults >18	6,927	3,671	1,432	39%
Green space	Quality of green space (pleasantness)	Life satisfaction	Adults >65	588	463	125	27%
Green space	Quality of green space (safety)	Life satisfaction	Adults >65	588	463	125	27%
Places to play	Park improvements	Park use	Men >18	3,616	2,134	-	0%
Total				9,241			

a Specific environmental change, intervention, asset or hazard).

b Cases expected in same population per annum without any environmental pathways applied.

c Baseline refers to net cases in present day scenario. (Attributable cases per annum minus expected cases).

Green cells indicate health improvement.

##### 3.2.5.2. Health benefits of green space at Frome Gateway: Future scenarios

The potential changes to health in cases under each scenario are compared in Table 3.

**Table 3:** Net attributable health risks and benefits affected by green space: Baseline and scenarios 1–3 compared.

**Environment change (intervention, asset or hazard)**	**Health outcome**	**Baseline cases^a^**	**S1 cases^b^**	**S2 cases^c^**	**S3 cases^d^**
NDVI increase	Cancer (mouth and throat)	-	−0.23	−0.23	−0.23
NDVI increase	Respiratory (asthma)	-	-	8	8
NDVI increase	Weight gain	-	-	−41	−41
Proximity to green space	Activity	2,632	2,632	2,632	2,632
Proximity to green space	Mental health	125	125	125	125
Proximity to green space	Respiratory (asthma)	5	5	5	5
Proximity to large, attractive, open space	Activity	1,432	1,432	1,432	1,432
Quality of green space (pleasantness)	Life satisfaction	125	125	125	125
Quality of green space (safety)	Life satisfaction	125	125	125	125
Size of public open spaces	Diabetes	-	-	−10	-
Park improvements	Park use	-	171	171	171
Park use	Diabetes	−3	−3	−3	−3
Park use	Weight gain	−127	−139	−139	−139

a Baseline scenario: Population 9,241, all other conditions as present day.

b Scenario 1: Population 9,241, Policy Compliant.

c Scenario 2: Population 9,241, 1Ha additional green space in single unit.

d Scenario 3: Population 9,241, 1Ha additional green space dispersed across site.

Red highlighted text denotes deterioration in health. Green text indicates improvement in health.

Improvements to park quality and safety are assumed to lead to increased park use in all scenarios: this has benefits in terms of reduced risk of diabetes and weight gain.

Scenarios 1–3 indicate a possible increase in NDVI which may lead to reduced risk of mouth and throat cancer. Scenarios 2 and 3, which have the largest potential for increases in NDVI score, indicate potential reductions in risk of being overweight or obese for children.

Greenness, estimated *via* NDVI, may continue to have an impact on increased risk of asthma in children, and this effect is seen in higher values for Scenarios 2 and 3 where there is the most potential for higher NDVI scores.

Health outcomes such as activity, wellbeing and life satisfaction identified in the baseline scenario are not shown to change under the three future scenarios, indicating that the threshold for these is already met by the existing provision of green space.

##### 3.2.5.3. Provision of a large park vs. small pocket parks

Only one change is unique for Scenario 2 compared with the other scenarios, and that relates to an increase in the size of public open spaces. The relevant impact-pathway is found to relate to cases of diabetes and has a specific threshold value of 0.7 hectares. In Scenario 2, the specific provision of an additional park of around 1 hectare unlocks this pathway, potentially leading to a reduction in 10 cases of diabetes from a baseline of 41 cases in the population considered here. Over 25 years, this could lead to savings in health valued at around £22.7 million. In Scenario 3, where additional green space is dispersed across the site, this threshold is not reached and these benefits are therefore not realized.

##### 3.2.5.4. Valuation of health effects over the lifetime of the project

**Figure 5:**
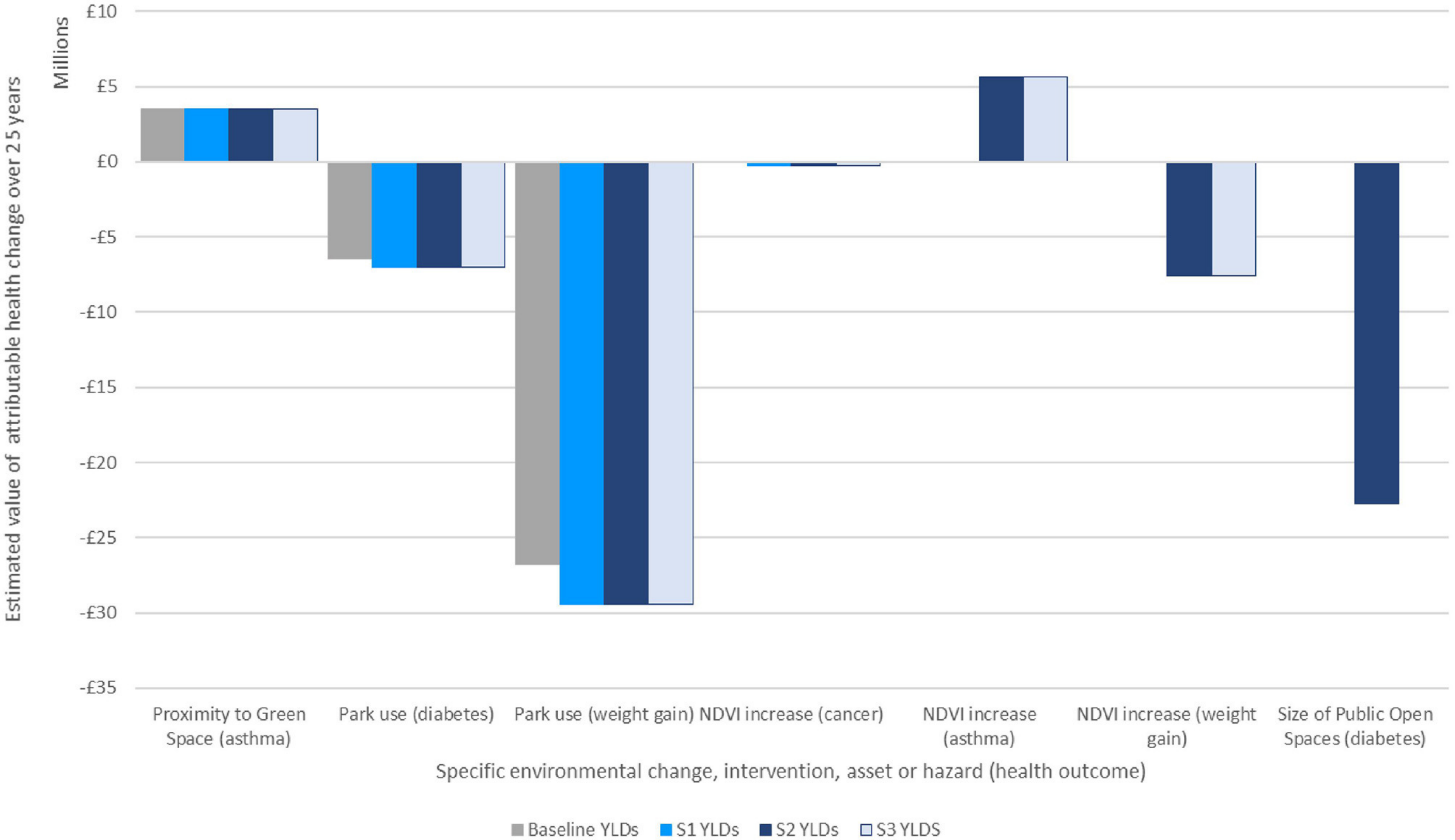
Estimate of economic valuation of attributable changes to morbidity over 25 years under each scenario.

Figure 5 indicates the potential value of attributable changes to morbidity by individual impact-pathways related to green space. This is the sum of the value of changes in years of illness over 25 years under each of the scenarios.

It may be helpful to summarize the total value of health changes by scenario: the figures below have not been adjusted for double counting, but to sum the total effect of each scenario in turn may help indicate the magnitude of the difference between them:

- Scenario 1 (Policy Compliant) is estimated to improve morbidity to a value of around £3.5 million above the baseline.
- Scenario 2 (Future Scenario with single additional open space) is estimated to improve health to a value of around £28 million above the baseline.
- Scenario 3 (Future Scenario with dispersed green space) is estimated to improve health to a value of £5.4 million.

#### 3.2.6. Sensitivity analysis

We have assumed that the highest change in NDVI score would be 0.105–0.15 points, which is not enough to reach the HAUS threshold for reductions in premature mortality. If the NDVI could be raised by 0.24 points for the site, we estimate that premature mortality might be reduced by 3 cases per year–equivalent to around 680 premature life years over the lifetime of the project, at a value of around £41.5 million. However, a change in NDVI on this scale represents a dramatic change in the land use at Frome Gateway, including considerably more tree cover, and may not be achievable or appropriate for this urban site given other ambitions such as provision of housing and business space.

## 4. Discussion

There is growing demand for new approaches that will enable us to better account for the social and environmental external costs in urban development. A range of public- and private-sector stakeholders face a significant challenge in how they interpret and respond to evidence on a wide range of external costs and navigate the conflicts that these may generate with competing development objectives.

This study brings together in a model a substantial account of the current evidence base relating to the quantification and economic valuation of health impacts associated with the urban environment. Societal costs of illness for 78 health outcomes are incorporated into a model that represents 28 characteristics of the built environment. This approach offers an evidence-led way of comparing the effects of different urban form elements in terms of the potential magnitude of impact on health.

The interviewee responses suggest that both public and private sector representatives appear to be aware of many of the major health challenges posed by poor-quality urban environments. However, interviewees also recognized that health is not factored adequately into the urban planning process. There appeared to be considerable support for greater use of economic valuation to help improve decision-making. More specifically, interviewees suggested a very wide range of potential leverage points at which this type of valuation might be fed into the urban development system, at national and local level [see interview findings in Black et al. (12), Figure 1]. It was recognized that there is no “silver bullet” solution, with quantitative valuation of health impacts just one possible mechanism amongst the range of interventions needed.

With regards to the green infrastructure modeling, the HAUS model highlights the important role that existing parks and green spaces have for the health of local people, as well as the potential health benefits of improving the quality and quantity of these spaces. However, it importance will only be clear to potential users if outputs are presented in easy-to-digest forms and in ways meaningful to them. The exercise also serves to emphasize the need to define and measure changes in the urban environment–in this case potential changes to NDVI scores for the site under different scenarios, which may be resource-intensive for the model user.

The strength of the HAUS tool lies in its capacity to synthesize evidence from two strands of literature–on health impact pathways and economic valuation of health impacts–and combine it in such a way that specific project- or policy-based changes in the urban environment can be evaluated against health-related criteria. At the same time, there are inherent challenges in synthesizing evidence from across such a wide range of urban health and economic valuation literature which itself is derived from a diverse range of empirical contexts, using contrasting methodologies, assumptions and reporting protocols. There are also wide divergences in the quantity and quality of evidence available across the range of environmental characteristics. For example, children's health forms an important component of costs when relating to air quality, noise and food environment. However, there is very limited evidence on child health in the economic valuation literature. The resulting health impacts are therefore likely to be significantly undervalued.

We have not explored fully here the extensive uncertainties which are clearly present, therefore, within every aspect of the modeling process and this may be thought to reduce the value of the tool outputs. However, there are significant uncertainties inherent to any form of economic valuation of health outcomes. Such evidence is nonetheless widely used across decision and policy making systems and is especially prevalent in areas that require significant investment, such as in health and urban infrastructure, where it is currently used to justify expenditure (24, 43). Thus, uncertainties in valuation, however sizeable, do not necessarily negate its' usefulness; this depends on how that information is understood, used and valued. Future development of this tool and comparable endeavors that address the need for health to be given adequate weighting in urban development processes therefore require substantial attention being given to how such data can be most effectively communicated to the full range of stakeholder types. At the same time, further research is needed to help fill the more sizeable gaps identified in the literature so that, for example, there is a re-balancing of the weight of evidence toward areas other than air pollution in both health quantification and health valuation.

## Data availability statement

The original contributions presented in the study are included in the article/Supplementary material, further inquiries can be directed to the corresponding author.

## Author contributions

EE: methodology, investigation, writing–original draft, and visualization. AH: conceptualization, methodology, supervision, and writing–review and editing. DB: conceptualization, writing–original draft, and writing–review and editing. All authors contributed to the article and approved the submitted version.
