# Supplementary material for: Developing and testing an environmental economics approach to the valuation and application of urban health externalities
Source: Front Public Health. 2023 Feb 17;11:1070200. doi: 10.3389/fpubh.2023.1070200 (PMC9982114; doi:10.3389/fpubh.2023.1070200)
Supplement: Supplementary file 1 [file Table_1.docx]

|  |  |  |  | HEALTH IMPACT Range | | HEALTH IMPACT Range | |  |
| --- | --- | --- | --- | --- | --- | --- | --- | --- |
| Grouping | Characteristic | Impact Pathway | Population | OR^[[1]](#endnote-1)^ Low | OR High | Alt^[[2]](#endnote-2)^ Low | Alt High | Specific Health outcome |
| Building design | Cold | Central Heating improvements > Respiratory | children 6-12s with asthma | 0.35 | 0.85 |  |  | Wheezing |
| Building design | Cold | Central Heating improvements > Sickness absence | children 6-12s with asthma | - | - | 0.11 | 3.13 | Fewer days off school |
| Building design | Cold | Central Heating lack > Mortality | Adults >65 | 1.01 | 1.02 |  |  | Mortality (all cause) |
| Building design | Cold | Improvement to thermal quality > Cardiovascular | Adults > 18 | - | - |  |  | Blood pressure changes |
| Building design | Cold | Insulation retrofit > GP/hospital | All | 0.67 | 0.84 |  |  | Hospital admissions |
| Building design | Cold | Insulation retrofit > Respiratory | Adults >60s | 1.30 | 1.66 |  |  | Respiratory symptoms |
| Building design | Cold | Insulation retrofit > Sickness absence | Adults > 18 | 0.46 | 0.83 |  |  | Fewer days off work |
| Building design | Cold | Insulation retrofit > Sickness absence | Children | 0.31 | 0.80 |  |  | Fewer days off school |
| Building design | Cold | Serious draught > Mental health | Adults > 18 | 1.41 | 3.69 |  |  | Mental health problems (psychological distress ) |
| Building design | Conditions | Living in flats > General health | Adults > 18 | 1.02 | 3.20 |  |  | Self rated health (risk of poor) |
| Building design | Conditions | Living in outlying areas > General health | Adults > 18 | 1.30 | 5.10 |  |  | Self rated health (risk of poor) |
| Building design | Conditions | Upgrade of housing quality > GP/hospital | Adults >65 | 0.53 | 0.83 |  |  | Hospital admissions |
| Building design | Damp | Damp home > Respiratory | Adults > 18 | 1.26 | 3.50 |  |  | Acute Respiratory Conditions |
| Building design | Damp | Damp home > Respiratory | Children | 1.69 | 7.18 |  |  | Asthma (Child) |
| Building design | Damp | Floor condition > Respiratory | Children 1-8 | 1.36 | 4.82 |  |  | Asthma (Child) |
| Building design | Indoor air quality | Cockroaches > GP/hospital | Children 0-13 with asthma | 1.63 | 14.63 |  |  | A&E visit |
| Building design | Indoor air quality | Cockroaches > Sleep disorder | Children 0-13 with asthma | 2.52 | 29.43 |  |  | Sleep disorder |
| Building design | Indoor air quality | Dust exotoxin level > Respiratory | Children 9-12 | 0.29 | 0.96 |  |  | Asthma (Child) |
| Building design | Indoor air quality | Floor type > Allergies | Children 0-13 | 1.13 | 2.09 |  |  | Eczema |
| Building design | Indoor air quality | Installation of ventilation system > Respiratory | Children with moderate or severe asthma | - | - | 2.80 | 11.40 | Asthma: Points on PedsQL asthma summary score |
| Building design | Indoor air quality | Mould > Headache | Children 0-13 with asthma | 1.19 | 14.80 |  |  | Headache |
| Building design | Indoor air quality | Mould > Respiratory | Vulnerable groups | 1.04 | 1.20 |  |  | Wheezing |
| Building design | Indoor air quality | Poor ventilation > Mortality | Children | 0.97 | 1.74 |  |  | Mortality |
| Building design | Indoor air quality | Respiratory hazards > Respiratory | Vulnerable groups | 1.01 | 1.22 |  |  | Asthma attacks |
| Building design | Overheating | Impact of heatwaves > Mortality | Adults >50 | 1.03 | 1.12 |  |  | Mortality (non-accidental) |
| Building design | Safety/ Accessibility for Vulnerable groups | Safety modifications > Injuries | All | 0.41 | 0.91 |  |  | Injuries (domestic) |
| Climate Change | Flooding | Flooding evacuation > PTSD | Adults > 18 | 1.69 | 4.83 |  |  | PTSD (PTSD of HTQ-R scale ≥2) |
| Climate Change | Flooding | Flooding evacuation+1yr > Anxiety | Adults > 18 | 1.30 | 2.93 |  |  | Anxiety (GAD-2 score)≥3 |
| Climate Change | Flooding | Flooding evacuation+1yr > Depression | Adults > 18 | 1.12 | 2.46 |  |  | Depression (PHQ-2) ≥ 3 |
| Climate Change | Flooding | Flooding evacuation+1yr > PTSD | Adults > 18 | 1.17 | 2.48 |  |  | PTSD (PCL-6 score)≥14 |
| Climate Change | Flooding | Flooding exposure > Anxiety | Adults > 18 | 3.00 | 7.80 |  |  | Anxiety (GAD-7 score)>10 |
| Climate Change | Flooding | Flooding exposure > Asthma | Adults > 18 | 1.20 | 4.40 |  |  | Asthma |
| Climate Change | Flooding | Flooding exposure > Depression | Adults > 18 | 1.60 | 4.30 |  |  | Depression (PHQ-9 Score ≥10) |
| Climate Change | Flooding | Flooding exposure > Earache | Adults > 18 | 1.10 | 4.10 |  |  | Ear Infections |
| Climate Change | Flooding | Flooding exposure > Psychological distress | Adults > 18 | 3.40 | 7.30 |  |  | Psychological distress (GHQ-12>3) |
| Climate Change | Flooding | Flooding exposure > PTSD | Adults > 18 | 1.90 | 7.80 |  |  | PTSD (PCL-6 score)≥14 |
| Climate Change | Flooding | Flooding exposure+0.5yr > Anxiety | Adults > 18 | 1.18 | 14.70 |  |  | Anxiety (GAD-2 score)≥3 |
| Climate Change | Flooding | Flooding exposure+0.5yr > Depression | Adults > 18 | 1.50 | 40.13 |  |  | Depression (PHQ-2) ≥ 3 |
| Climate Change | Flooding | Flooding exposure+0.5yr > PTSD | Adults > 18 | 3.91 | 53.13 |  |  | PTSD (PCL-6 score)≥14 |
| Climate Change | Flooding | Flooding exposure+1yr > Anxiety | Adults > 18 | 3.77 | 11.24 |  |  | Anxiety (GAD-2 score)≥3 |
| Climate Change | Flooding | Flooding exposure+1yr > Depression | Adults > 18 | 3.91 | 10.99 |  |  | Depression (PHQ-2) ≥ 3 |
| Climate Change | Flooding | Flooding exposure+1yr > PTSD | Adults > 18 | 4.33 | 11.93 |  |  | PTSD (PCL-6 score)≥14 |
| Climate Change | Flooding | Flooding exposure+2yr > Anxiety | Adults > 18 | 1.70 | 16.30 |  |  | Anxiety (GAD-2 score)≥3 |
| Climate Change | Flooding | Flooding exposure+2yr > Anxiety/ Depression | All | 4.60 | 13.50 |  |  | Anxiety/ Depression |
| Climate Change | Flooding | Flooding exposure+2yr > Depression | Adults > 18 | 1.09 | 39.80 |  |  | Depression (PHQ-2) ≥ 3 |
| Climate Change | Flooding | Flooding exposure+2yr > Pain/ discomfort | All | 1.50 | 4.20 |  |  | Pain/ discomfort |
| Climate Change | Flooding | Flooding exposure+2yr > Usual activities | All | 2.50 | 11.90 |  |  | Usual activities |
| Climate Change | Flooding | Flooding exposure+3yr > Anxiety/ Depression | All | 2.50 | 7.70 |  |  | Anxiety/ Depression |
| Climate Change | Flooding | Flooding exposure+3yr > Pain/ discomfort | All | 1.50 | 6.10 |  |  | Pain/ discomfort |
| Climate Change | Flooding | Flooding exposure+3yr > Usual activities | All | 1.50 | 4.20 |  |  | Usual activities |
| Climate Change | Flooding | Storm or flood exposure > Common mental disorder | Adults >16 | 1.08 | 2.07 |  |  | Common mental disorder |
| Climate Change | Overheating | Increase in mean daily temperature > GP/hospital | Adults >75 | 1.04 | 1.18 |  |  | Hospital admissions (respiratory) |
| Climate Change | Overheating | Increase in mean daily temperature > Suicide | All | 1.02 | 1.06 |  |  | Mortality (Suicide) |
| Community infrastructure | Fast food outlets | Density/ Accessibility of fast food outlets > Diabetes | All singleton pregnancies | 1.21 | 2.19 |  |  | Gestational diabetes |
| Community infrastructure | Food environment | Unhealthy food environment > Diet | Adults > 18 | 1.03 | 1.22 |  |  | risk of abdominal obesity |
| Community infrastructure | Fruit &Veg access | Access to fruit and vegetables > Diet | Adults > 18 |  |  | 1.01 | 1.09 | Increase in amount of F&V intake |
| Community infrastructure | Fruit &Veg access | Healthy food environment > Weight gain | All | - | - |  |  | Units BMI |
| Community infrastructure | Places to Play | Gyms & Recreation centres > Activity | Adults > 18 | 3.52 | 14.95 |  |  | activity ≥150min/week |
| Community infrastructure | Places to Play | Park improvements > Park use | Men > 18 |  |  |  |  | First time park users |
| Community infrastructure | Places to Play | Park quality/ features > Activity | Children | - | - | 2.30 | 8.10 | Bouts of 15 mins activity per week |
| Community infrastructure | Places to Play | Park use > Diabetes | Adults >35 | 0.58 | 0.90 |  |  | Diabetes Mellitus |
| Community infrastructure | Places to Play | Park use > Weight gain | Adults >35 | 0.64 | 0.84 |  |  | Obesity |
| Community infrastructure | Small stores | Specialist food stores nearby > Weight gain | Adults > 18 | 0.45 | 0.74 |  |  | Obesity |
| Community infrastructure | Walkability | New walking infrastructure > Activity | Adults > 18 | - | - | 6.50 | 24.20 | minutes per week walking & cycling per kilometre |
| Community infrastructure | Walkability | Quality of paths to NOS > Activity | Adults>65 | 1.03 | 3.74 |  |  | Higher levels of walking (>2.5 hours per week) |
| Community infrastructure | Walkability | Walkability rating > Activity | Adults 18-65 | 1.49 | 2.55 |  |  | Walking (transport) |
| Community infrastructure | Walkability | Walkability rating > Diabetes | Adults > 18 | 0.80 | 0.97 |  |  | Diabetes Mellitus |
| Community infrastructure | Walkability | Walkability rating > Mental health | Men >65 | 0.12 | 0.82 |  |  | Depression |
| Community infrastructure | Walkability | Walkability rating > Weight gain | Women >50 | 0.88 | 1.00 |  |  | Overweight/ Obesity |
| Community infrastructure | Within walking distance | Proximity to shops > Activity | Women >18 | 0.52 | 4.72 |  |  | Any walking occasion |
| Natural environment | Air quality | Air pollution (mixed components) > Mortality | Adults>25 | 1.07 | 1.08 |  |  | Mortality |
| Natural environment | Air quality | Air pollution (mixed components) > Weight gain | children 5-11 |  |  |  |  | Units BMI increase at age 10 |
| Natural environment | Air quality | Benzene > Ear Infections | children 12-18 months | 0.93 | 1.46 |  |  | Ear Infections |
| Natural environment | Air quality | Benzene > Respiratory (LRTI) | children 12-18 months | 0.94 | 1.19 |  |  | LRTI |
| Natural environment | Air quality | Black Carbon > Allergies | Children <7 | 1.04 | 2.75 |  |  | Eczema |
| Natural environment | Air quality | Black Carbon > GP/hospital | Adults>40 | 1.00 | 1.03 | 0.01 | 0.05 | Increase in CHD hospitalisation |
| Natural environment | Air quality | Black Carbon > GP/hospital | Adults>40 |  |  | 0.02 | 0.10 | Increase in COPD hospitalisation |
| Natural environment | Air quality | Black Carbon > Mortality | Adults 25-59 | 1.03 | 1.10 |  |  | Non-accidental mortality |
| Natural environment | Air quality | CO concentration > Allergies | Infants <1 | 1.06 | 1.78 |  |  | Eczema |
| Natural environment | Air quality | CO concentration > Parkinson's Disease | Adults >60s | 1.06 | 1.21 |  |  | Parkinson’s Disease |
| Natural environment | Air quality | NO/NO2 concentration > Cancer (brain) | Adults >50 | 1.25 | 4.19 |  |  | Cancer (brain) |
| Natural environment | Air quality | NO/NO2 concentration > Cancer (breast) | Women >18 | 1.05 | 1.67 |  |  | Cancer (breast) |
| Natural environment | Air quality | NO/NO2 concentration > Cancer (cervical) | Women >50 | 1.01 | 5.93 |  |  | Cancer (cervical) |
| Natural environment | Air quality | NO/NO2 concentration > Cancer (lung) | Adults >35 | 1.03 | 1.07 |  |  | Cancer (lung) |
| Natural environment | Air quality | NO/NO2 concentration > Cancer (skin) | Adults > 18 | 1.16 | 1.34 |  |  | Cancer (NMSC) |
| Natural environment | Air quality | NO/NO2 concentration > Diabetes | Women >18 | 1.16 | 1.73 |  |  | Diabetes Mellitus |
| Natural environment | Air quality | NO/NO2 concentration > Ear Infections | Infants <2 | 1.00 | 1.34 |  |  | ENT infections |
| Natural environment | Air quality | NO/NO2 concentration > LRTI | Infants <2 | 0.98 | 1.12 |  |  | LRTI |
| Natural environment | Air quality | NO/NO2 concentration > Mortality | Adults > 40 | 1.03 | 1.08 |  |  | Mortality (all cause) |
| Natural environment | Air quality | NO/NO2 concentration > Parkinson's Disease | Adults >60s | 1.03 | 1.16 |  |  | Parkinson’s Disease |
| Natural environment | Air quality | NO/NO2 concentration > Respiratory (bronchitis) | Infants <1 | 1.03 | 1.66 |  |  | Bronchitis |
| Natural environment | Air quality | NO2 concentration > Allergies | Children <10 | 0.73 | 0.97 |  |  | Aeroallergen sensitization |
| Natural environment | Air quality | O3 concentration > Allergies | Children <10 | 1.02 | 1.64 |  |  | Allergic Rhinitis |
| Natural environment | Air quality | O3 concentration > Diabetes | Infants <1 | 1.04 | 2.35 |  |  | Type 1 Diabetes |
| Natural environment | Air quality | O3 concentration > Mortality | Adults >25 | 1.01 | 1.03 |  |  | Non-accidental mortality |
| Natural environment | Air quality | PM10 concentration > Cancer (breast) | Women > 18 | 1.09 | 1.31 |  |  | Cancer (breast) |
| Natural environment | Air quality | PM10 concentration > Cancer (mouth and throat) | Adults > 18 | 1.31 | 1.78 |  |  | Cancer (Mouth and Throat) |
| Natural environment | Air quality | PM10 concentration > Cancer (prostate) | Men > 18 | 1.08 | 1.39 |  |  | Cancer (Prostate) |
| Natural environment | Air quality | PM10 concentration > Cancer (skin) | Adults > 18 | 1.35 | 1.72 |  |  | Cancer (NMSC) |
| Natural environment | Air quality | PM10 concentration > Cardiovascular | Adults > 18 | 1.05 | 2.16 |  |  | Cardiovascular disease |
| Natural environment | Air quality | PM10 concentration > Child development | Newborns | 1.03 | 1.61 |  |  | Child Development |
| Natural environment | Air quality | PM10 concentration > Diabetes | Adults > 18 | 1.17 | 1.67 |  |  | Diabetes Mellitus |
| Natural environment | Air quality | PM10 concentration > Mortality |  | 1.02 | 1.06 |  |  | Mortality (all cause) |
| Natural environment | Air quality | PM2.5 concentration > Mortality | Adults>40 | 1.02 | 1.06 |  |  | Mortality |
| Natural environment | Air quality | PM2.5 concentration > Respiratory (asthma) | Children <2 | 1.11 | 1.61 |  |  | Cough without infection |
| Natural environment | Air quality | PM2.5 concentration > Respiratory (ENT) | Children <2 | 1.00 | 1.27 |  |  | ENT infections |
| Natural environment | Air quality | PM2.5 concentration > Respiratory (Flu) | Children <2 | 1.00 | 1.27 |  |  | Flu |
| Natural environment | Air quality | PM2.5 concentration > Stroke | Adults > 60 | 1.05 | 1.87 |  |  | Stroke incidence |
| Natural environment | Air quality - Industrial | Air pollution (mixed components) > Allergies | Infants <1 | 1.10 | 1.94 |  |  | Eczema |
| Natural environment | Air quality - Industrial | Landfill emissions > GP/hospital | Women >18 | 1.00 | 1.09 |  |  | Hospital admissions (CVD) |
| Natural environment | Air quality - Industrial | PM10 concentration > Mortality | Adults > 18 | 1.03 | 1.90 |  |  | Mortality (Pancreatic Cancer) |
| Natural environment | Air quality - Industrial | Proximity to Cement Factory > GP/hospital | People with chronic CVD/ respiratory disease | 1.61 | 9.01 |  |  | Hospital admissions |
| Natural environment | Air quality - Industrial | Sox/ SO2 exposure > GP/hospital | Women >18 | 1.01 | 1.27 |  |  | Hospital admissions (respiratory) |
| Natural environment | Air quality - Industrial | Sox/ SO2 exposure > Mortality | Adults >40 | 1.50 | 1.10 |  |  | Mortality (all cause) |
| Natural environment | Green space | Access to Public Open Space > Activity | Adults 18-60 | 1.06 | 2.13 |  |  | Higher levels of walking |
| Natural environment | Green space | Garden access > Activity | All | 1.15 | 2.05 |  |  | Odds of meeting PA guidelines |
| Natural environment | Green space | Garden ownership > Wellbeing | All | 1.02 | 1.47 |  |  | evaluative wellbeing |
| Natural environment | Green space | Green space: Agricultural > Activity | Adults > 18 | - | - | -0.13 | -0.04 | Less Time spent cycling (Beta) |
| Natural environment | Green space | Green space: Agricultural > Mental health | Adults > 18 | 0.86 | 0.97 |  |  | Depression |
| Natural environment | Green space | Green space: Urban > Activity | Adults > 18 | - | - | 0.01 | 0.25 | More Time spent cycling (Beta) |
| Natural environment | Green space | Green space: Urban > Mental health | Adults > 18 | 1.00 | 1.26 |  |  | Depression |
| Natural environment | Green space | NDVI > Cancer (mouth and throat) | Adults > 18 | 0.83 | 0.96 |  |  | Cancer (Mouth and Throat) |
| Natural environment | Green space | NDVI > Mortality | Adults >35 | 0.94 | 0.96 |  |  | Non-accidental mortality |
| Natural environment | Green space | NDVI > Respiratory | Children <7 | 1.10 | 1.85 |  |  | Asthma (Child) |
| Natural environment | Green space | NDVI > Weight gain | Children 9-12 | 0.81 | 0.87 |  |  | Overweight/ Obesity |
| Natural environment | Green space | Proximity to Green Space > Activity | Adults > 18 | 1.18 | 4.35 |  |  | Odds of Activity (150mins p/w) v Inactive (no or v low activity) |
| Natural environment | Green space | Proximity to Green Space > Activity | Pregnant women |  |  | 2.66 | 15.62 | Mins MVPA per day (beta) |
| Natural environment | Green space | Proximity to Green Space > Mental health | Adults >65 | 1.16 | 4.06 |  |  | Life satisfaction |
| Natural environment | Green space | Proximity to Green Space > Respiratory (asthma) | Children 9-12 | 1.09 | 2.36 |  |  | Asthma (Child) |
| Natural environment | Green space | Proximity to Large, attractive, Open Space > Activity | Adults > 18 | 1.08 | 1.79 |  |  | Walking (sufficient >150 mins per week) |
| Natural environment | Green space | Quality of Green Space (Pleasantness) > Life satisfaction | Adults >65 | 1.03 | 3.63 |  |  | Life satisfaction |
| Natural environment | Green space | Quality of Green Space (Safety) > Life satisfaction | Adults >65 | 1.04 | 3.54 |  |  | Life satisfaction |
| Natural environment | Green space | Quality of Green Space (Serenity) > Mental health | Women > 18 who are physically active | 0.06 | 0.09 |  |  | Risk of poor mental health (measured via GHQ12) |
| Natural environment | Green space | Size of Public Open Spaces > Diabetes | Adults > 18 | 0.69 | 0.83 |  |  | Diabetes Mellitus |
| Natural environment | Green space | Visiting Blue Space > General health | Adults > 50 | 1.10 | 2.60 |  |  | Wellbeing (Average-High) |
| Natural environment | Proximity to main road | Proximity to main road > Allergies | Newborns | 1.01 | 1.51 |  |  | Allergic Rhinitis |
| Natural environment | Proximity to main road | Proximity to main road > Diabetes | Women >18 | 1.03 | 1.27 |  |  | Diabetes Mellitus |
| Natural environment | Proximity to main road | Proximity to main road > Mental health | Adults > 18 | 1.06 | 1.08 |  |  | Dementia |
| Natural environment | Proximity to main road | Proximity to main road > Respiratory | All | 1.03 | 1.14 |  |  | Breathlessness |
| Socio-economics | Affordability | Prolongued exposure to affordability problems > Mental health | Adults >16 |  |  | -2.18 | 0.49 | Mental Health score (Beta) (measured using the SF-36 MCS). |
| Socio-economics | Affordability | Transition to unaffordable > General health | Adults >16 |  |  | -0.02 | -0.10 | Self rated health (change in avg) |
| Socio-economics | Affordability | Transition to unaffordable > Mental health | Adults 25-64 (home purchasers, lowest 40% income bracket) |  |  | -0.03 | -0.94 | Mental Health score: Tenure-specific estimated mean change in GHQ |
| Socio-economics | economic status of area | Neighbourhood deprivation > Mental health | Adults >16 | 1.08 | 1.28 |  |  | Psychological distress (GHQ12>1) |
| Socio-economics | economic status of area | Relocation to area of lower deprivation > Functional loss | Adults >16 | 1.15 | 1.71 |  |  | Functional health limitations (>0 limitations) |
| Socio-economics | economic status of area | Relocation to area of lower poverty > Mental health | Adults > 18 |  |  | 0.80 | 0.80 | Depressive symptoms (% reduction) |
| Socio-economics | economic status of area | Socio-economic status > Weight gain | Men >55 | 0.80 | 0.91 |  |  | Obesity |
| Socio-economics | Fear of crime | Inadequate lighting > Functional loss | Adults >55 | 1.36 | 7.56 |  |  | Self-reported functional loss (NAGI index) |
| Socio-economics | Fear of crime | Perceptions of neighbourhood > Weight gain | Adults > 18 | 0.77 | 0.94 |  |  | Obesity |
| Socio-economics | Fear of crime | Perceptions of safety > Activity | Women >18 | 0.31 | 0.38 |  |  | Walking (any reported occasion) |
| Socio-economics | Fear of crime | Problems with neighbourhood environment > Functional loss | Adults >55 | 1.15 | 8.51 |  |  | Lower-Extremity Functional loss |
| Socio-economics | Fear of crime | Refraining from going out > General health | Adults >16 | 1.24 | 1.36 |  |  | Less than good SRH |
| Socio-economics | Fear of crime | Refraining from going out > Mental health | Adults >16 | 1.39 | 1.54 |  |  | Poor psychological wellbeing (GHQ12) |
| Socio-economics | Regeneration | Neighbourhood renewal > General health | Adults >18 in disadvantaged communities | 1.23 | 3.74 |  |  | Improved general health |
| Socio-economics | Tenure | Home ownership v renting > General health | Adults >20 | 1.26 | 1.47 |  |  | Poor SR health (dichotomous Very Poor or Poor v Very good-Fair) |
| Socio-economics | Tenure | Long term social housing exposure > Weight gain | Adults >55 |  |  | 46.40 | 58.60 | %Classified as obese (BMI>30), not an OR |
| Transport | Cycling infrastructure | Conversion of intersections to roundabouts > Injuries | All (cyclists) |  |  | 0.27 | 0.27 | Accidents with injuries |
| Transport | Cycling infrastructure | Traffic free cycling infrastructure > Activity | Adult cycle commuters | 1.03 | 1.76 |  |  | Cycling commuting time increase |
| Transport | Noise | Noise (general) > Functional loss | Adults >55 | 1.38 | 5.30 |  |  | Self reported functional loss (NAGI index) |
| Transport | Noise | Traffic noise > Activity | Adults > 18 | 0.94 | 1.00 |  |  | MVPA>150mins |
| Transport | Noise | Traffic noise > Anti-social behaviour | School aged children 5-6 years | 1.04 | 2.38 |  |  | Child Conduct problems |
| Transport | Noise | Traffic noise > Diabetes | Adults 50-64 | 1.05 | 1.18 |  |  | Diabetes Mellitus |
| Transport | Noise | Traffic noise > Mental health | Men aged 45-59 | 1.21 | 3.24 |  |  | Psychological ill health |
| Transport | Noise | Traffic noise > Mental health (child) | School aged children 5-6 years | 1.04 | 2.72 |  |  | Child Emotional problems |
| Transport | Noise | Traffic noise > Mortality | Men > 18 | 1.00 | 1.04 |  |  | Mortality (MI) |
| Transport | Noise | Traffic noise > Mortality | Women >18 | 1.01 | 1.03 |  |  | Mortality (Hypertension) |
| Transport | Noise | Traffic noise > Sleep disorder | Adults >19 |  |  | 1.04 | 1.18 | Sleep disturbance (discrete difference in % reporting) |
| Transport | Public transport links | Availability of public transport > Mental health | Several groups | 0.87 | 0.98 |  |  | Prescription of anti-depressants |
| Transport | Road Safety | Driveway design > Injuries | Children <7 | 0.40 | 3.00 |  |  | Injuries (RTA) |
| Transport | Road Safety | Section control > Injuries | All |  |  | 0.51 | 0.51 | Fatalities or serious injuries RTA |
| Transport | Road Safety | Speed reducing infrastructure > Injuries | All | 0.42 | 0.57 |  |  | Injuries (RTA) |
| Transport | Road Safety | Traffic signals > Injuries | All | 0.43 | 0.99 |  |  | Injuries (RTA) |

1. OR: Change in health observed in terms of Odds Ratio or Risk Ratio [↑](#endnote-ref-1)
2. ALT: Alternative measure of health change [↑](#endnote-ref-2)
