# Supplementary material for: Developing and testing an environmental economics approach to the valuation and application of urban health externalities
Source: Front Public Health. 2023 Feb 17;11:1070200. doi: 10.3389/fpubh.2023.1070200 (PMC9982114; doi:10.3389/fpubh.2023.1070200)
Supplement: Supplementary file 2 [file Table_2.docx]

Supplementary material Table 2: Societal Costs of Illness (UK)

| **Health outcome** | **Direct** | **Indirect** | **Disutility** | **TOTAL** | **Range Low** | **Range High** |
| --- | --- | --- | --- | --- | --- | --- |
| Activity (Benefit) | - 4,589 | -3,698 | -3,499 | -11,785 | -5,821 | -18,044 |
| Alcohol misuse | 267 | 2,518 | 2,455 | 5,240 | 5,192 | 5,240 |
| Allergies: atopic dermatitis (child) | 112 | 6 | 359 | 477 | 477 | 477 |
| Asthma (adult) | 239 | 171 | 1,605 | 2,016 | 1,599 | 3,974 |
| Asthma (child) | 233 | 120 | 2,327 | 2,680 | 1,890 | 3,855 |
| Birth defects (VLBW) | 26,024 | 9,764 | 6,984 | 42,772 | 32,401 | 42,772 |
| Birth defects (CHD) | 26,703 | 1,932 | 9,825 | 38,460 | 38,460 | 38,460 |
| Cancer (brain) male | 19,388 | 36,122 | 18,906 | 74,416 | 45,115 | 66,876 |
| Cancer (brain) female | 21,746 | 36,122 | 18,906 | 76,774 | 47,471 | 69,235 |
| Cancer (Breast) (pre-menopausal) | 16,757 | 13,075 | 16,699 | 46,532 | 40,456 | 55,448 |
| Cancer (Breast) (post-menopausal) | 16,757 | 2,822 | 16,699 | 36,278 | 32,949 | 42,447 |
| Cancer (cervical) | 18,497 | 4,792 | 2,236 | 25,524 | 25,517 | 25,532 |
| Cancer Colorectal (male) | 18,506 | 1,599 | 18,906 | 39,011 | 38,561 | 48,026 |
| Cancer Colorectal (female) | 17,641 | 1,599 | 18,906 | 38,146 | 37,694 | 47,163 |
| Cancer Kidney (male) | 13,714 | 10,697 | 18,906 | 43,316 | 37,978 | 59,348 |
| Cancer Kidney (female) | 14,158 | 11,043 | 18,906 | 44,107 | 38,566 | 59,101 |
| Cancer Leukaemia (Child) | 103,119 | - | 12,825 | 115,944 | 111,969 | 119,917 |
| Cancer (Lung) | 9,480 | 1,441 | 4,962 | 15,883 | 11,748 | 25,053 |
| Cancer (Mouth and Throat) (male) | 16,801 | 13,039 | 18,906 | 48,746 | 42,205 | 64,739 |
| Cancer (Mouth and Throat) (female) | 16,717 | 13,105 | 18,906 | 48,728 | 42,165 | 64,742 |
| Cancer (Skin) | 5,086 | 9,196 | 18,906 | 33,188 | 33,187 | 42,641 |
| Cardiovascular disease (year1) | 11,653 | 4,100 | 2,875 | 18,627 | 6,768 | 37,574 |
| Cardiovascular disease (stable) | 3,518 | 4,100 | 2,875 | 10,493 | 2,455 | 18,531 |
| Conduct (child) | 281 | - | - | 281 | 281 | 281 |
| Cycling (benefit) | -4,589 | -3,698 | -3,493 | -11,785 | -5,821 | -18,044 |
| Day off school | - | 108 | 132 | 240 | 240 | 240 |
| Day off work | - | 108 | 65 | 173 | 173 | 173 |
| Dementia | 15,659 | 18,916 | 17,266 | 51,841 | 43,208 | 60,474 |
| Depression | 6,759 | 21,611 | 21,611 | 49,980 | 39,175 | 60,785 |
| Depressive complaints | 6,759 | 21,611 | 21,611 | 49,980 | 39,175 | 60,785 |
| Depressive symptoms | 6,759 | 21,611 | 21,611 | 49,980 | 39,175 | 60,785 |
| Depressive symptoms (Pregnant women) | 6,759 | 24,108 | 29,927 | 60,794 | 60,794 | 60,794 |
| Depression medication | 6,759 | 21,611 | 21,611 | 49,980 | 39,175 | 60,785 |
| Diabetes | 3,565 | 2,947 | 1,489 | 8,001 | 7,083 | 35,929 |
| Diet: Low Fruit & Veg intake | 32 | 106 | - | 138 | 138 | 138 |
| Eczema (Child) | 112 | 6 | 359 | 477 | 477 | 477 |
| Emotional distress | 4,276 | 19,223 | 11,750 | 35,249 | 29,374 | 41,124 |
| Emotional symptoms (child) | 147 | - | - | 147 | 147 | 147 |
| GP visit | 39 | - | - | 39 | 39 | 39 |
| Headache | 648 | 3,226 | 22 | 3,896 | 3,874 | 11,995 |
| Hospital admissions (average) | 3,293 | - | 4,628 | 7,921 | 2,404 | 13,930 |
| Hospital admissions (CHD) | 1,295 | - | 4,628 | 5,923 | 1,917 | 102,024 |
| Hospital admissions (CVD) | 1,162 | - | 4,628 | 5,790 | 1,917 | 102,024 |
| Hospital admissions (respiratory) | 1,109 | - | 4,628 | 5,737 | 1,966 | 65,585 |
| Hospital admissions (COPD) | 1,490 | - | 4,628 | 6,118 | 2,270 | 14,904 |
| Hypertension | 462 | 298 | - | 760 | 760 | 760 |
| Injuries (domestic) | 1,472 | 4,660 | 12,264 | 18,396 | 16,433 | 20,480 |
| Injuries (RTA) | 3,554 | 18,230 | 54,484 | 76,268 | 30,544 | 1,354,188 |
| Mental health problems | 3,366 | 3,905 | 3,635 | 10,907 | 9,089 | 12,724 |
| Mortality (Value of a Statistical Life) | 1,198 | 697,582 | 1,330,457 | 2,029,237 | 1,014,619 | 3,246,779 |
| Mortality (Value of a Life Year) | # | # | # | 61,019 | 30,509 | 97,630 |
| Obesity (adult) | 78 | 215 | 467 | 761 | 727 | 795 |
| Obesity (child) | 212 | - | 458 | 670 | 24 | 1,754 |
| Obesity: BMI increases Adult | 5 | - | 93 | 99 | - 42 | 218 |
| Obesity: BMI increases Adult | 160 | 215 | 467 | 843 | 686 | 1,000 |
| Obesity: BMI increases Adult | 307 | 215 | 935 | 1,457 | 1,267 | 1,649 |
| Obesity: BMI increases Adult | 501 | 215 | 1,402 | 2,118 | 1,894 | 2,344 |
| Overweight/ Obesity (adult) | 5 | - | 93 | 99 | - 42 | 218 |
| Overweight/ Obesity (child) | 212 | - | 458 | 670 | 24 | 1,754 |
| Parkinson's Disease (1st year) | 6,389 | 29,779 | 126 | 36,294 | 36,294 | 36,294 |
| Parkinson's Disease (Subsequent years) | 8,071 | 38,144 | 126 | 46,341 | 46,341 | 46,341 |
| Respiratory: Acute Respiratory Conditions | - | - | 54 | 54 | - | 2,157 |
| Respiratory: Breathlessness | - | - | 11 | 11 | - | 539 |
| Respiratory: Bronchitis (Acute) child | # | # | # | 361 | 234 | 398 |
| Respiratory: Cough (Child) | 19 | 336 | 20 | 375 | 376 | 395 |
| Respiratory: Dry cough at night | 119 | 1,163 | 2,615 | 3,897 | 3,960 | 4,709 |
| Respiratory: Ear Infection (Infant) | 198 | 670 | 111 | 979 | 915 | 1,091 |
| Respiratory: Flu (Infant) | 106 | 400 | 39 | 545 | 506 | 8,395 |
| Respiratory: LRTI | 19 | 336 | 20 | 375 | 376 | 395 |
| Sleep disorders | 119 | 1,163 | 2,615 | 3,897 | 3,960 | 4,709 |
| Stress, anxiety, or depression | 4,276 | 19,223 | 11,750 | 35,249 | 29,374 | 41,124 |
| Stroke (1st year after Incidence) | 19,491 | 29,460 | 16,009 | 64,960 | 61,344 | 68,576 |
| Stroke (subsequent years) | 8,364 | 18,347 | 16,009 | 42,720 | 37,821 | 47,619 |
| Hospital admissions (A&E) | 166 | - | 4,628 | 4,794 | 1,822 | 10,332 |
| Walking (benefit) | - 4,589 | -3,698 | -3,493 | -11,785 | - 5,821 | -18,044 |
| Wellbeing | - | - | 13,000 | 13,000 | 10,000 | 16,000 |
|  |  |  |  |  |  |  |

# Estimated societal costs of illness in the UK, per year, GBP2019.
