# Supplementary material for: Developing and testing an environmental economics approach to the valuation and application of urban health externalities
Source: Front Public Health. 2023 Feb 17;11:1070200. doi: 10.3389/fpubh.2023.1070200 (PMC9982114; doi:10.3389/fpubh.2023.1070200)
Supplement: Supplementary file 3 [file Data_Sheet_1.PDF]

UPSTREAM
Interviewer Field Notes

	Does this evidence influence your thinking?	How could this evidence change practice?	How might you use this data?	Is there any more information you would find useful?
**Interviewee 1 (Public Sector)**	• Reinforces mostly (e.g. GI, buildings) – much needed!<br>• Increasing awareness of mental health<br>• Most surprised by: noise on children's behaviour, poor air quality/productivity (and related valuation)	• Strategic: Treasury > Green Book (5-stage business case)<br>• Housing Infrastructure Funding (bits of kit)<br>• Making the case for quantifying<br>• LPAs<br>• Policy<br>• Local plan inspector<br>• Quantifying evidence in planning<br>• Guidance for local authorities (TCPA climate change)	• OOPR<br>• Evidence base for local plan<br>• Health advisor<br>• RTPI<br>• Reference in conversation to members<br>• Health work (e.g. dementia and practice advice)<br>• What stops us creating healthier place? (blog)	• Aggregated valuation important<br>• Wales Value of Planning £2.3bn for Welsh Economy<br>• £ vs life expectancy
**Interviewee 2 (Public Sector)**	• Well understood: Cold, Air quality – UoLeicester; Green space / mental health<br>• Noise a surprise; surprised too not to see overheating affecting medical costs and productivity; also indoor air quality scale of issue surprising	• Targeted – evidence base to support local planning > understanding more about problem<br>• Building regs > overheating + cold (MHCLG)<br>• Air quality, strategic view o old housing/regeneration<br>• Design Briefs for bigger sites – (Satellite Catapult)<br>• End Users not landlords > max flexibility for the occupier > high employment so competitive > national organisations (e.g. Google attraction)	• Policy > Local Plan<br>• Sustainable Transport – outcomes<br>• HIA<br>• Hot food takeaways<br>• Brokering and partnerships > Jointly commissioned work with developers<br>• County transport<br>• CCG	• PHE Evidence base used in policy
**Interviewee 3 (Public Sector)**	• All expected<br>• Dementia/air qual a surprise + mental health/noise (not there)<br>• Interesting so many relating to car<br>• Proximity to main road in 'natural environment'?<br>• Quantification useful – extent to which some more important than others (rather than £s)	• Bit late for JSP/JTP – via WECA and N.Somerset<br>• Central Govt. should be interested in NHS costs and productivity…indoor air quality and GI – via campaign groups and think tanks?	• Local Plan Review – housing density; quality indicators; 1m2 amenity space; GLA (consultation) – feed in via senior officers/cabinet members<br>• Council as Developer: design – via architects + Housing Delivery Team<br>• Air quality consultation – measures<br>• One City Plan/Approach – building relationships; range of agencies<br>• Fuel Poverty/warm homes strategy – JSNA, HWB<br>• Public don't determine, though can influence – where is the call to action? • It will arm some activist citizens and campaign groups; public don't weigh up options/info – more about gut instinct (e.g NIMBY's)	• Noise not from neighbours – building design/acoustics? Other people vs traffic?

Supplementary Material Table 3: Interview Field Notes

UPSTREAM
Interviewer Field Notes

**Interviewee 4 (Public Sector)**	• Broadly not surprising (e.g. respiratory linked to cold)<br>• Useful to see health/wellbeing linking to productivity and cost<br>• Most surprised by noise linking to transport, and economic status<br>• Strange not to see: fast food to weight gain; dementia linked to air quality	• ROI cases > addressing quality of home environment Taking a place-based approach<br>• Cost of productivity > LEP > District Councils (productivity and growth)<br>• National > DCLG, DoH, PHE<br>• STPs, HWB Boards<br>• Growth Board Deal > County, District, CCG, LEP > 100,000 homes, national funding, Healthy New Towns (April 2019)	• Productivity!<br>• 3 Pathway LEPs<br>• Industrial Strategy<br>• Healthy new Towns – Phase II (town-wide)<br>• July Partnership (Strategic, Academic, 3rd Sector)	• Understanding noise<br>• Causal pathways and associations<br>• Cold > embarrassed > mental health
**Interviewee 5 (Public Sector)**	• Very interesting – good to see set out like this<br>• Know about green space and mental health<br>• Noise/child conduct disorder, overheating and indoor air quality new	• Green space and air quality already high on the agenda<br>• Interesting to see noise and indoor air quality as significant or higher than those, which could support GLA and national government<br>• Best via public health officers to feed in to planners	• Interim chief exec mentioned noise<br>• Mitigate particularly from rail given residential development<br>• Good for increasing justification within masterplanning<br>• So far main work on severance, contamination, level changes - engineering	• Overall burden<br>• Incidence very low<br>• Overall population exposure
**Interviewee 6 (Public Sector)**	• Air quality and access to GI known<br>• Noise is interesting – people just accept that it's there without questioning impact<br>• Can't comment on medical aspects	• Need to simplify for development industry<br>• Policy/requirements needed – building regs alone for e.g. acoustics – NPPF not saying much on either<br>• Air quality currently high on the political agenda (after affordable housing)<br>• Noise could be moved up the agenda<br>• Planning? Railway/road noise impacts – could see that an increasing issue<br>• Needs to sign to cost savings for NHS..<br>…which could pay for advice, ability, skill-sets	• Air qual and GI already doing<br>• Noise could easily be integrated in to our thinking<br>• It's no leap of faith - core mission is "how do you create great spaces/places"<br>• Challenge is cost – NHS? GI? Productivity?<br>• Helps tell the story (e.g. to investors, tenants, etc.)	

UPSTREAM
Interviewer Field Notes

**Interviewee 7 (Private Sector)**	• Almost surprised it's not more. Bears out my assumptions.	• As a lever in policy debate?<br>• Who owns the issue? Starting point to find out who responsible. Complex process – web of contributory agencies. Which bit is causing the problem? E.g. Road: DfT? Mending Roads? Car manufacturers? Citizens? Public Policy / wicked problem	• Finding investment; housing projects; social/environmental, diversity, community<br>• We would want to take in to account 'pain and suffering' costs<br>• Character areas as a checklist possibly<br>• Costs less useful, though could help with prioritisation<br>• Part of pitch to partners possibly<br>• Costs are externalities so more to do with wider narrative than straight cost-benefit…even NHS does not benefit from prevention!<br>• Costs perhaps best presented to Whitehall/Bank of England with regards productivity, (which could perhaps filter down in to better standards?)	• Social infrastructure/resilience; community; space to gather/chat?<br>• Holistic view of quality of place<br>• Qualitative more than quant perhaps<br>• Difficult to show causality?<br>• Social spaces?
**Interviewee 8 (Private Sector)**	• Noise is new – double glazing?<br>• Fast food so low?<br>• Known: building design; damp/cold; crime/socio-economic; air qual	• NHS property sell off – get that wrong? – externalities could be integrated in – land disposal<br>• Education and messaging – property sector, NHS, policy-makers; information session and graphics needed	• How we allocated investment<br>• Criteria for housing sites – transport, socio-econoimc<br>• North Bristol – broker (funding); long-lease holder, tenancy agreements – how sold – social property fund	• Exec summary<br>• Colour confusing
**Interviewee 9 (Private Sector)**	• All worth unpacking<br>• Some too macro – what influence has it had?<br>• Gyms, fast food, green space well known – and indoor air quality<br>• But links to dementia and child conduct disorder new<br>• May change thoughts on noise<br>• Numbers useful	• BREEAM / Well Standard (latter more science based) – credits tweaked / criteria<br>• Commercial / communities<br>• Building Regs / London Plan<br>• New-build (BCO; CIBSE; ICE; CIRIA)<br>• Existing the challenge – might it be used in e.g. land ownership, maintenance, LPAs?	• Some high end developers, yes, but others?<br>• UKGBC/ULI – sound bites lead to industry discussion (e.g. productivity (temperature a 5-10% increase)<br>• No body to translate<br>• Internal socio-economic valuation of proposals	• Socio-economic<br>• HACT – standard benchmarks – for every m2 – what about quality/sensitivity? Badly designed vs high?

UPSTREAM
Interviewer Field Notes

**Interviewee 10 (Private Sector)**	• Most definitely<br>• Most obvious, but dementia surprising<br>• Sums involved useful – scale of difference<br>• Overheating is very current for us, particularly new properties and in this weather! London in particular…	• Noise – planning regs already factor in nr major roads<br>GI – premium in premium place (lower densities, higher qual of open space) – harder for e.g. Persimmon<br>Suburban vs urban debate – externalities – electric vehicles (noise and emissions)	• Place-making Design Principles – NHS Healthy New Towns Network (participant) – Homes Plus? – Ebbsfleet DC<br>• Social Value – 2 surveys (return 1,000 from 8,000) – 'discreet choice experiments'* – proximity to open space – what value? – consumer-led demand – intuitively people know, but what's the £? – this we use to engage re: planning and negotiations with local stakeholders. – Based on Green Book?<br>• Already doing socio-economic (e.g. jobs, value added)<br>* e.g. Lexus – pay for nicer features – how much will people pay?; health costs from limited exercise and insurance	
**Interviewee 11 (Private Sector)**	• Child conduct and dementia surprising<br>• Not surprised by socio-economics and all the rest	• Building regulations – revisions – e.g noise from traffic (vehicle vs building) – sound insulation, barriers<br>• GI/mental health – planning issue (we would do some – if level playing field)<br>• Socio-economic – what can we do?<br>• Risk of overheating more than cold for new build<br>• Indoor air quality – VOCs<br>• BREEAM Communities – attractive places – env/econ/phys – planting street trees	• Could help as a checklist when considering Building for Life, BREEAM Comms, Secured by Design – integrating within tools	
**Interviewee 12 (Private Sector)**	• £ big numbers!<br>• Road safety not a problem<br>• Noise interesting! Is it road or internal noise? Only noise complaints in our developments have been apartments; acoustics standards – random testing passed. Older people complain…higher densities?	• Regulatory: 1) buildings regs + 2) planning (green space, air qual)<br>• Anything in NPPF on noise?<br>• Only major objections relate to transport, highways, congestion and (increasingly) air quality	• Secured by Design – Building for Life<br>• Cold no longer an issue<br>• Selling homes: middle to upper end (families ½ of market so health and wellbeing high)<br>• Marketing – yes<br>• Not when buying land…possibly measures to mitigate noise from transport (1/4 acre site – blocks of flats – little to do)<br>• Larger sites – scale important – inner city more difficult (no open space)<br>• 'Suburban' not rural	

UPSTREAM
Interviewer Field Notes

**Interviewee 13 (Private Sector)**	• Not really<br>• Narrow areas that don't feature…perhaps in social housing (e.g. cold is not applicable to us)<br>• Large, neighbourhood-scale projects (2-3,000 units – e.g. Taunton) do need to consider wider aspects, such as green space, transport, etc., but most developments not at this scale…even up to 2-400<br>• It's a fairly shallow list (of considerations) – I could think of perhaps 100 more items	• Don't think it should or will	• I wouldn't	
**Interviewee 14 (Private Sector)**	• Depends on assumptions<br>• Noise (scale of) surprising<br>• Overheating – nice to see crystalised<br>• Green space – obvious<br>• Add substance to mental health	• Public sector – will reduce burden on you<br>• Might accept different value on land<br>• Disconnect between health and built environment<br>• Must be at Chief Exec level – impossible lower down due to dislocation<br>• Same in Govt departments<br>• NHS Trust, LPA<br>• "best consideration" – usually financial	• If solid, via stories<br>• Brief in specifying product<br>• Promotion of discussion with public<br>• Plan-making<br>• Thoughtful, better informed<br>• Good aide-memoir<br>• Vision statement<br>• Prioritisation and agenda-setting	

UPSTREAM
Interviewer Field Note - Acronyms

Acronyms	
BCO	British Council for Offices
BREEAM	Building Research Establishment Environmental Assessment Method
CCG	Clinical Commissioning Group
CIBSE	Chartered Institution of Building Services Engineers
CIRIA	Construction Industry Research and Information Association
DCLG	Department for Communities and Local Government
DfT	Department for Transport
DoH	Department of Health
GI	Green Infrastructure
HACT	Housing Associations Charitable Trust
HIA	Health Impact Assessment
HWB	Health and Wellbeing Board
ICE	Institute of Civil Engineers
JSNA	Joint Strategic Needs Assessment
JSP/JTP	Joint Spatial/Transport Plan
LEP	Local Enterprise Partnership
LPA	Local Planning Authority
MHCLG	Ministry of Housing Communities and Local Government
NHS	National Health Service
NIMBY	Not In My Back Yard'
OOPR	Old Oak and Park Royal
PHE	Public Health England
RTPI	Royal Town Planning Institute
STP	Sustainability and Transformation Plan
TCPA	Town and Country Planning Association
UKGBC	UK Green Building Council
ULI	Urban Land Institute
VOCs	Volatile Organic Compounds
WECA	West of England Combined Authority
