# Supplementary material for: Developing and testing an environmental economics approach to the valuation and application of urban health externalities
Source: Front Public Health. 2023 Feb 17;11:1070200. doi: 10.3389/fpubh.2023.1070200 (PMC9982114; doi:10.3389/fpubh.2023.1070200)
Supplement: Supplementary file 4 [file Data_Sheet_2.PDF]

HEALTH MAP	PHE Topics	Vancouver Healthy Toolkit	BREEAM Communities	HUDU Rapid HIA	Egan Review
**Global Ecosystem**					
Climate Stability	Natural & Sustainable Environment	Environmentally sensitive areas<br>Urban heat island	Transport carbon emissions	Climate change	Environmental
Biodiversity	Natural & Sustainable Environment	Environmentally sensitive areas	Ecology strategy<br>Enhancement of ecological value	Nature	Environmental
**Natural Environment**					
Natural Habitats	Natural & Sustainable Environment	Access Natural Environment<br>Environmentally sensitive areas<br>Natural Environments<br>Open space	Green infrastructure	Open space<br>Nature	Environmental
Air	Natural & Sustainable Environment	Air pollution<br>Environmentally sensitive areas<br>Natural Environments	Microclimate	Air quality<br>Nature	Environmental
Water	Natural & Sustainable Environment	Environmentally sensitive areas<br>Natural Environments	Flood risk assessment / management<br>Rainwater harvesting<br>Water pollution<br>Water strategy	Nature	Environmental
Land	Natural & Sustainable Environment	Existing communities<br>Environmentally sensitive areas<br>Land use<br>Natural Environments	Land use<br>Landscape	Open space<br>Nature	Environmental

Supplementary material Table 4: Urban Form Characteristics

**Built Environment**						
	Buildings	Housing	Adequate housing for all<br>Affordable housing<br>Diverse types and tenures<br>Homeless<br>Housing<br>Housing for marginalised groups<br>Location of housing	Existing buildings and infrastructure<br>Housing provision<br>Local vernacular<br>Sustainable buildings<br>Community management of facilities<br>Utilities<br>Low impact materials	Housing<br>Healthcare	Housing
	Places	Neighbourhood Design	Compact neighbourhoods<br>Urban heat island<br>Neighbourhood Design<br>Transportation Networks	Design review<br>Layout<br>Light pollution<br>Noise pollution<br>Public realm<br>Community management of facilities	Healthcare<br>Noise	The Built Environment
	Streets	Neighbourhood Design	Connectivity<br>Safe networks<br>Transportation Networks	Safe and appealing streets		Transport & Connectivity
	Routes	Neighbourhood Design	Connectivity<br>Public transit<br>Safe networks<br>Transportation Networks	Cycling network		Transport & Connectivity
**Activities**						
	Working	Neighbourhood Design	Connectivity	Labour and skills		Economy
	Shopping	Neighbourhood Design	Connectivity	Delivery of services, facilities and amenities		Economy<br>Services

	Moving	Transport	Active travel<br>Attractive road, rail and waterways<br>Connectivity<br>Convenient<br>Mobility<br>Public transit<br>Safe networks<br>Transportation Networks	Access to public transport<br>Cycling facilities & network<br>Local parking<br>Public transport facilities<br>Transport assessment<br>Transport carbon emissions	Active Travel<br>Access	Transport &<br>Connectivity<br>Services
	Living	Neighbourhood Design<br>Transport	Connectivity	Delivery of services, facilities and amenities<br>Utilities	Crime<br>Healthcare<br>Noise	Social & Cultural<br>Services
	Playing	Neighbourhood Design	Connectivity	Delivery of services, facilities and amenities		Social & Cultural<br>Services
	Learning	Neighbourhood Design	Connectivity	Delivery of services, facilities and amenities		Services
**Local Economy**						
	Wealth creation			Economic impact	Employment	Economy
	Resilient markets					Economy<br>Services
**Community**						
	Social capital		Elderly<br>Existing communities<br>Safety	Inclusive design<br>Consultation	Social cohesion<br>Care for elderly<br>Crime	Social & Cultural
	Social networks		Connectivity	Demographic needs and priorities	Social Infrastructure	Social & Cultural
**Lifestyle**						
	Diet/nutrition	Food	Access to healthy food<br>Agricultural capacity<br>Community-scale food infrastructure<br>Food Systems		Food	

	Work-life balance			Delivery of services, facilities and amenities		
	Physical activity	Neighbourhood Design<br>Transport	Connectivity<br>Walkability<br>Safe networks	Cycling network		

Topics missing from Health Map*	Adapting to climate chang	Use of resources*	Governance***
	Energy strategy***	Crime****	
	Low impact materials***		
	Resource efficiency***		

** N.B. This does not infer whether one form of appraisal is more comprehensive than any other; rather that the starting list of topic areas is fuller. For example, while BREEAM Communities has more criteria than any of the others, the criteria are prescribed and narrow in definition - usually one or a small number of individual aspects - while the topic areas from the other are wholely reliant on interpretation. See written text on this subject for a fuller analysis.*

*** Though, like many other criteria, it's not written explicitly, adapting to climate change could be interpreted under any/all of the Health Map*

**** These are related to urban form, but their impact on health tends to be indirect through global implications of energy and resource use (e.g. deforestation, climate change). Arguably however, the impact of low impact materials in housing and offices (e.g. volatile organic compounds in paint) can have direct impact on health, hence its inclusion in the 'buildings' section.*

***** These are critical to health, but not elements of urban form*
